# Nanohydrogel Composite Vaccine Capable of Bypassing the Blood–Brain Barrier and Targeting Tumors for Glioblastoma Immunotherapy via Intranasal Immunization

**DOI:** 10.3390/vaccines14090797

**Published:** 2026-09-10

**Authors:** Dawei Dai, Shuo Han, Guangming Wang, Yongming Qiu, Ang Li

**Affiliations:** 1 Department of Neurosurgery, Ren Ji Hospital, Shanghai Jiao Tong University School of Medicine, Shanghai 200127, China; daidawei@renji.com; 2 Department of Neurosurgery, Changzheng Hospital, Naval University School of Medicine, Shanghai 201209, China; hanshuo@sjtu.edu.cn; 3 Department of Neurosurgery, Shanghai Geriatric Medical Center, Shanghai 200032, China; wang.guangming@zsgmc.sh.cn; 4 School of Life Science and Technology, Tongji University, Shanghai 200092, China

**Keywords:** glioblastoma, nanohydrogel vaccine, intranasal immunization, blood–brain barrier

## Abstract

**Background**: Glioblastoma (GBM) is a primary malignant tumor of the central nervous system and has a high lethal rate despite therapeutic advances. Although immunotherapies have achieved great success in solid tumors, the highly immunosuppressive tumor microenvironment and the blood–brain barrier (BBB) obstruction hinder the development of immunotherapies for GBM. In this study, we innovatively developed a nanohydrogel composite vaccine (nanoCOM-GEL) for GBM immunotherapy via intranasal immunization. **Methods**: The nanoCOM-GEL used GelMA as the hydrogel matrix and was co-formulated with antigenic peptides, the BBB-penetrating peptide (peptide 22), as well as immune cell stimulants and chemokines. The efficiency of this vaccine in bypassing the BBB and its capacity to induce anti-GBM immune responses were evaluated in vitro and in vivo. **Results**: The nanoCOM-GEL vaccine demonstrated superior BBB-bypassing and BBTB-penetrating capabilities, potent immunostimulatory activity, and effective GBM-targeting efficacy, as validated in both cellular and animal models. In an orthotopic GBM mouse model (*n* = 8 per group), intranasal immunization with nanoCOM-GEL significantly extended median survival from 21 days (control group) to more than 60 days (nanoCOM-GEL group), representing a 2.8-fold increase (*p* < 0.001). The vaccine markedly inhibited tumor growth, as evidenced by an 82.5% reduction in tumor tissue at day 21 compared to controls (*p* < 0.01). Mechanistically, nanoCOM-GEL increased intratumoral CD8^+^ T cell infiltration by 9.5-fold and upregulated DCs by 6.4-fold, while simultaneously reducing intratumoral M2-type tumor-associated macrophages by 79.3% (*p* < 0.001), effectively reshaping the immunosuppressive tumor microenvironment. **Conclusions**: This innovative nanoCOM-GEL vaccine achieved potent anti-GBM therapeutic efficacy and provided a promising strategy for effective GBM immunotherapies.

## 1. Introduction

Glioblastoma multiforme (GBM) is the most aggressive and lethal primary brain malignancy in adults, characterized by rapid progression and poor prognosis [1]. The achievement of immunotherapy in various solid tumors such as lung cancer, breast cancer, and melanoma has brought new hope for the treatment of GBM [2]. However, the efficacy of immunotherapy for GBM is poor due to the following reasons: the physical barrier of the BBB and a highly immunosuppressive tumor microenvironment (TME) [3,4]. The BBB strictly restricts therapeutic antibodies and vaccine to enter the brain, which is a major hurdle for GBM immunotherapy. Meanwhile, the immunosuppressive microenvironment of GBM restricts activation of effector T cells [5,6]. The tumor-derived metabolites (such as kynurenine) further reinforce immune tolerance through the aryl hydrocarbon receptor signaling pathway [7,8].

In recent decades, the nose-to-brain delivery strategy has gained significant attention due to its unique advantages in bypassing the BBB and avoiding systemic clearance [9,10]. Anatomically, there are direct pathways between the nasal cavity and the brain through which nanoparticles can enter the central nervous system. Recent studies have shown that a biomineralized nanovaccine based on hepatitis B core virus-like particles (HBc VLPs) exhibited significant accumulation in glioma tissues via intranasal administration [11]. Another study also showed that intranasal delivery of a survivin peptide-CpG nanovaccine could accumulate in cervical lymph nodes and tumor sites through nasal mucosal lymphatic drainage and the olfactory bulb/trigeminal nerve pathways [12]. These studies have suggested the application of nanovaccines via nose-to-brain delivery in GBM immunotherapy.

Although nose-to-brain delivery has significant advantages, there are also some unfavorable factors that reduce its delivery efficiency, such as mucosal clearance, epithelial barrier and limited retention time. Previous studies have shown that hydrogels based on GelMA (gelatin methacryloyl) have excellent biocompatibility and the ability for controlled drug release. The GelMA hydrogel structure protects fragile drugs (mRNA, peptides) from enzymatic degradation and improves antigen half-life. The mucosal adhesion property of GelMA further prolongs the nasal retention time, which enhances the ability of GelMA nanohydrogel to enter the brain via the olfactory/trigeminal nerve pathways. Furthermore, the RGD motifs on the nanohydrogel surface are designed to promote integrin-mediated binding and retention at the tumor site and facilitate intratumoral immunity [13,14].

Furthermore, the nanovaccine still needs to traverse the blood–brain tumor barrier (BBTB) to reach GBM cells after reaching the brain. To enhance the ability of the nanohydrogel vaccine to penetrate the BBTB and target tumor cells after reaching the brain, we incorporated peptide 22 into the vaccine. Peptide 22 is a ligand for the low-density lipoprotein receptor (LDLR), which is overexpressed on brain capillary endothelial cells (BCECs) [15]. Zhang et al. demonstrated that LDLR is overexpressed in both brain capillary endothelial cells (BCECs) and glioma cells, and that peptide-22-decorated nanoparticles significantly increased cellular uptake in these cells. This enhanced uptake was significantly inhibited by excess free peptide-22, confirming the specificity of the LDLR-mediated pathway [16]. The same study also showed that peptide-22 decoration increased the transport ratio across an in vitro BBTB model and enhanced in vivo glioma targeting in intracranial glioma mouse models [17].

**Scheme 1:**
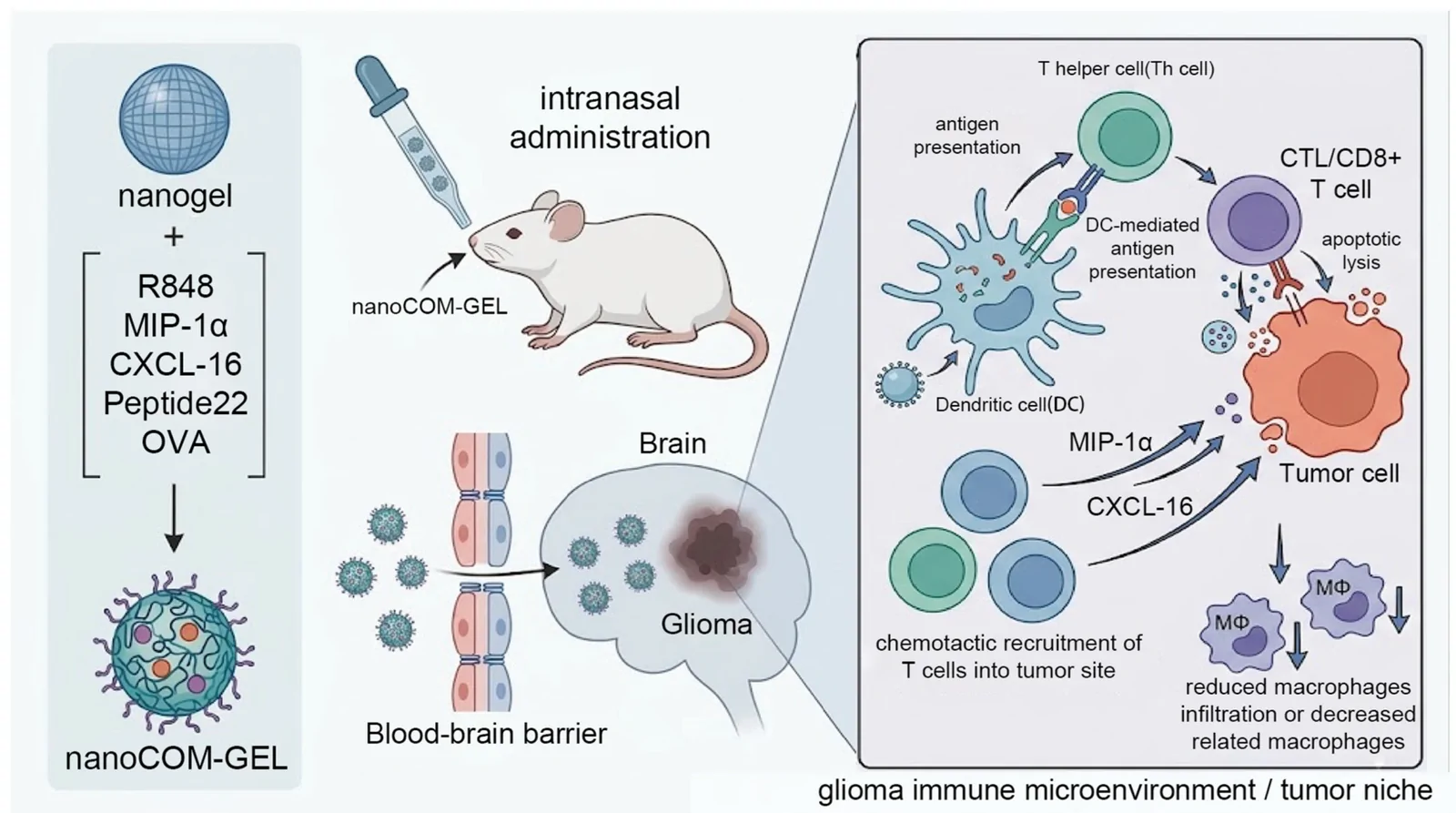
Schematic illustration of the accumulation of nanoCOM-GEL in GBM via intranasal administration and activation of the anti-GBM immune response.

In this work, we developed a compound GelMA loaded with both the OVA antigen and a BBB-crossing peptide (peptide 22), which is termed as nanoCOM-GEL. Meanwhile, we loaded nanoCOM-GEL with R848 to stimulate dendritic cell (DC) maturation [18] and immune cell chemokines (CXCL-16 and MIP-1α) to promote migration of immune cells to the tumor [19,20]. In GL261-OVA-luc orthotopic GBM mouse model, intranasal administration of nanoCOM-GEL could recruit T cells and DCs into the tumor, reverse the immunosuppressive microenvironment, elicit a robust antitumor immune response and stabilize a long-lasting immune response (Scheme 1). Our study highlights the advantages of the nanoCOM-GEL vaccine in enhancing nose-to-brain delivery efficiency and anti-GBM immunity, which will enable a broad range of mucosal vaccine applications against GBM.

## 2. Materials and Methods

### 2.1. Materials

The OVA peptides (257–264; 323–339) and peptide 22c (MPRLRGC) were purchased from GL Biochem (Shanghai, China) Ltd. The GL261-OVA-luc mouse GBM cell line was purchased from Zhongqiaoxinzhou Bio Company (Shanghai, China) and cultured in DMEM (Gibco, Suzhou, China) supplemented with 10% fetal bovine serum (FBS, Gibco, China), 100 U/mL penicillin, and 100 μg/mL streptomycin at 37 °C in a 5% CO_2_ incubator. Cells were passaged every 2–3 days upon reaching 80–90% confluence.

GelMA (EFL-GM-60) and GelMA lysis buffer were purchased from Engineering for Life (EFL, Suzhou, China). R848, CXCL16 protein, MIP-1α, GM-CSF, and IL-4 were purchased from MedChemExpress (MCE, Shanghai, China). Methanol and acetonitrile were purchased from Titan Technology Co., Ltd. (Shanghai, China) and used as received without further purification. The CD80-FITC antibody, CD86-APC antibody, CD40-PE antibody, MHC-II-PE-Cy7 antibody, CD8-FITC antibody, IFN-γ-PE antibody, permeabilization buffer, and IC fixation buffer were purchased from Invitrogen (Frederick, MD, USA). Transwell chambers were purchased from Sigma-Aldrich (St. Louis, MO, USA).

### 2.2. Animal Experiments

Six- to eight-week-old female C57BL/6 mice were purchased from Charles River Laboratories (Shanghai, China). The housing environment was controlled at 22–24 °C, 50–60% relative humidity, with a 12 h light/dark cycle. All animal procedures received approval from the Institutional Animal Care and Use Committee of Tongji University. For surgical procedures, anesthesia was induced with 5% isoflurane in oxygen and maintained at 2% via a nose cone. Buprenorphine (0.05–0.1 mg/kg, subcutaneous) was administered pre-operatively for analgesia and repeated every 8–12 h for 48 h post-operatively. Mice were monitored daily for body weight, neurological signs, and overall health status. Humane endpoints were defined as >20% body weight loss, severe neurological deficits (e.g., hunched posture, paralysis, or lethargy), or tumor burden exceeding ethical limits, at which point animals were humanely euthanized by cervical dislocation under deep isoflurane anesthesia (5% induction).

Mice were randomly assigned to treatment groups (*n* = 8 per group) using a computer-generated random number sequence. All animal procedures, including tumor inoculation, treatment administration, tumor monitoring, and tissue collection, were performed by investigators blinded to group allocation. Tumor volumes were measured using bioluminescence imaging, and the images were analyzed by an operator blinded to the treatment groups. Sample codes were unblinded only after the completion of all data collection and analysis. Predefined exclusion criteria included: (i) failure to establish a detectable tumor (BLI signal below threshold or tumor volume < 10 mm^3^) at day 7 post-inoculation; (ii) animals reaching humane endpoints (>20% body weight loss, severe neurological deficits, or tumor volume exceeding 10% of body weight); and (iii) accidental death unrelated to tumor progression (e.g., anesthesia-related causes). No animals were excluded from the final analysis unless they met these predefined criteria.

### 2.3. Preparation of NanoCom-Gel Vaccine

First, 0.05 g of lithium phenyl-2,4,6-trimethylbenzoylphosphinate (LAP) was precisely weighed and dissolved in 20 mL of phosphate-buffered saline (PBS) at 45 °C to prepare a 0.25% (*w*/*v*) solution. Subsequently, 1 g of GelMA (EFL-GM-60, EFL-Tech Co., Ltd., Suzhou, China) was accurately weighed and dissolved in 10 mL of the above 0.25% LAP solution under light-protected conditions at 65–70 °C to obtain the hydrogel precursor solution. The final concentrations of encapsulated components were: OVA peptide (257–264; 323–339) at 2 mg/mL, peptide 22 (MPRLRGC) at 200 μg/mL, R848 at 100 μg/mL, MIP-1α at 1 μg/mL, and CXCL16 at 1 μg/mL. After the temperature decreased to 38 °C, these components were added to the GelMA solution containing the photoinitiator. The mixture was injected into a continuous oil phase (mineral oil containing 5% Span 80 as the surfactant) to form water-in-oil (W/O) monodisperse droplets via microfluidics. Droplet size was controlled by adjusting the flow rate of the aqueous phase (10–50 μL/min) and oil phase (100–500 μL/min). The collected droplets were placed at 4 °C for 30 min to stabilize the droplet morphology, followed by blue light irradiation (405 nm, 10 mW/cm^2^) for 15 s to achieve partial photo-crosslinking. The oil phase and surfactant were then removed by centrifugation (14,000 rpm, 5 min) and washing with PBS three times. The size distribution of the nanoCOM-GEL was determined by dynamic light scattering (DLS) using a Zetasizer Nano ZS instrument (Malvern Panalytical, Worcestershire, UK). The zeta potential analysis was performed with a folded capillary cell (DTS1070, Malvern Panalytical). The abbreviations of nanogel vaccines with different components and their corresponding compositions are shown in Table S1.

### 2.4. NanoCOM-GEL Vaccine Release Assay In Vitro

The nanoCOM-GEL vaccine was co-cultured with GL261-OVA-luc cells in a 6-well plate (10^5^ cells/well) for 24 h. The supernatants were collected at four-hour intervals. The release of CXCL16 and MIP-1α was quantified using ELISA kits (ExCell Bio, Shanghai, China), and the cumulative release percentage was calculated using the formula: Cumulative release percentage = M/N × A × 100% (where M denotes the released amount, N the total drug loading, and A the dilution factor). For R848 release analysis, high-performance liquid chromatography (HPLC) was utilized. The quantification of R848 was performed using a high-performance liquid chromatography (HPLC) system equipped with a reversed-phase C18 column (Agilent Zorbax SB-C18, 3.5 µm, 75 × 4.6 mm, Agilent Technologies, Santa Clara, CA, USA) maintained at 40 °C. The mobile phase A (98% water/2% acetonitrile/0.1% trifluoroacetic acid) and mobile phase B (90% acetonitrile/10% water/0.09%) were delivered at a flow rate of 1.0 mL/min. A gradient elution program was applied as follows: 5% B at 0 min, linearly increased to 45% B at 7 min, then ramped to 95% B at 9 min, and finally returned to 5% B at 13 min for re-equilibration. The eluate was monitored by UV detection at a wavelength of 254 nm, with a typical retention time of approximately 3.5 min for R848. The standard curve of R848 was made with the concentration range from 0.1 µg/mL to 8 µg/mL; the concentrations of R848 in samples collected at various time points were determined with the standard curve.

### 2.5. NanoCOM-GEL Vaccine Degradation Test In Vitro

To evaluate the in vitro degradation behavior of the nanoCOM-GEL vaccine, hydrogel samples were prepared as described above and weighed to record the initial wet weight (W_0_). Briefly, pre-weighed nanoCOM-GEL samples (approximately 100 mg each) were placed in 5 mL of phosphate-buffered saline (PBS, pH 7.4) and incubated at 37 °C with gentle shaking at 50 rpm in a thermostatic shaker. At designated time points (days 1–11, every day), the samples were removed from the incubation medium, gently blotted with filter paper to remove surface water, and weighed to record the wet weight at each time point (W_t_). The degradation rate was calculated as the percentage of weight loss using the formula: Degradation (%) = [(W_0_ – W_t_)/W_0_] × 100%.

### 2.6. NanoCOM-GEL Vaccine Dimensional Stability Factor Detection

The nanoCOM-GEL droplets were placed on slides and then exposed to blue light for 15 s for solidification. Following solidification, the size of nanoCOM-GEL was measured and the volume (V) was calculated. At the same time, nanoCOM-GEL was immersed in sterile PBS. The size was measured every 12 h. The dimensional stability factor was determined using the formula (%) = [(Vf – Vi)/Vi] × 100. Vf = volume final and Vi = volume initial.

### 2.7. Culture of Bone Marrow-Derived Dendritic Cells (BMDCs) and Macrophages (BMDM)

Six-to-eight-week-old female C57BL/6 mice were sacrificed by decapitation under deep anesthesia. The femoral and tibial cavities were then flushed with sterile PBS, and the suspension was passed through a 40 μm cell strainer. After centrifugation at 1500 rpm for 5 min, the harvested bone marrow cells (10^7^ cells) were seeded into 3 mL of complete RPMI 1640 medium (Gibco) supplemented with IL-4 (10 ng/mL) and GM-CSF (20 ng/mL) and incubated at 37 °C. On day 3, the medium was gently removed and the cells were rinsed with sterile PBS. The 3 mL fresh medium containing the same concentrations of IL-4 and GM-CSF was added on days 3 and 5. Finally immature BMDCs were collected on day 7 for subsequent experiments. For culture of BMDM, the harvested bone marrow cells were resuspended in Dulbecco’s Modified Eagle Medium (DMEM, Gibco) supplemented with 10% FBS and mouse M-CSF (20 ng/mL) for 5 days. Then the suspended cells were removed and the adhered macrophages were collected for further research. To evaluate macrophage polarization, the macrophages treated with various hydrogel vaccines were harvested and detected the M1 or M2 related genes by RT-PCR.

### 2.8. DC and T Cell Migration Assay

To evaluate the chemotactic activity of DCs and T cells in response to the vaccine-released chemokines, transwell migration assays were performed using 24-well plates with polycarbonate membrane inserts (pore size 5 μm for DCs and 3 μm for T cells; Corning, Canton, NY, USA). Briefly, 5 × 10^4^ bone marrow-derived dendritic cells (BMDCs) or 1 × 10^5^ CD3^+^ T cells isolated from mouse splenocytes (using a magnetic bead-based negative selection kit; Miltenyi Biotec, San Jose, CA, USA) were resuspended in 100 μL of serum-free RPMI 1640 medium and seeded into the upper chamber. The lower chamber was filled with 600 μL of conditioned medium collected from the nanoCOM-GEL release study (containing released CXCL16 and MIP-1α), while medium collected from blank nanogel served as a negative control. After incubation at 37 °C in a 5% CO_2_ humidified atmosphere for 6 h, the cells that had migrated to the lower surface of the membrane were fixed with 4% paraformaldehyde for 15 min, stained with 0.1% crystal violet for 20 min, and gently rinsed with PBS. Non-migrated cells on the upper surface were carefully removed with a cotton swab. The stained cells on the lower membrane were imaged under an inverted light microscope (Nikon Eclipse Ti, 200× magnification, Sakae-ku, Nagaodai-Cho, Japan), and the numbers of migrated cells were counted in five randomly selected fields per well. All assays were performed in triplicate, and the chemotactic index was calculated as the fold-change in migrated cell number relative to the negative control group.

### 2.9. Dendritic Cell Maturation Detection Assay

The DCs were harvested according to the protocol described earlier and seeded into medium containing 10 ng/mL IL-4, 20 ng/mL GM-CSF and various nanohydrogel vaccines as indicated in the figure for 48 h. After incubation, the cells were washed with PBS and DC maturation was subsequently detected. For DC maturation analysis, antibodies were used as follows: PerCP-Cy5.5-CD11c, FITC-CD80, APC-CD40, FITC-CD86, and APC-MHC II antibodies (Thermofisher, Frederick, MD, USA). Isotype-matched control antibodies were used as negative controls. A minimum of 10,000 events were acquired per sample on a CytoFLEX flow cytometer (Beckman Coulter, Indianapolis, IN, USA), and data were analyzed using FlowJo VX software (V10). Gating strategy: cells were first gated on FSC/SSC to exclude debris, followed by gating on CD11c^+^ cells, and then analyzed for the expression of co-stimulatory molecules.

### 2.10. Establishment of an Orthotopic Glioma Model and Therapeutic Immunization Procedures

Mice were deeply anesthetized by intraperitoneal injection of 3% chloral hydrate anesthetic (anesthesia dose: 100 μL/10g body weight), combined with subcutaneous buprenorphine (0.05–0.1 mg/kg) for pre- and post-operative analgesia. After the mice were deeply anesthetized, the head was fixed in a stereotaxic apparatus, ensuring the incisor bar and ear bars were stable. The hair on the mouse head was partially shaved, and the surgical site was disinfected with alcohol. The scalp was incised along the midline to expose the skull and the skull surface was cleaned. Then, the zero point was set at the intersection of the coronal and sagittal sutures on the midline of the mouse brain (the bregma). At AP −0.4 mm, ML −1.5 mm relative to bregma, a mark was made and a burr hole was drilled using a cranial drill. The micro-syringe containing GL261-OVA-luc cells was moved to the burr hole and slowly lowered to a depth of DV −3.5 mm. The cell suspension (1 × 10^7^ cells/mL) was injected slowly (1 μL/min) (4 μL per mouse). Following injection, the needle was left in place for 5 min and withdrawn slowly. Then, the scalp incision was sutured. All surgical procedures were performed under aseptic conditions, and mice were monitored until fully recovered on a warm pad. After surgery, the mouse was placed on a warm bedding pad until fully awake. Four days after tumor cell inoculation (chosen to allow tumor engraftment before initiating treatment), tumor-bearing mice were randomly assigned using a computer-generated random number sequence to three groups (*n* = 8 per group, based on power analysis to detect a 30% difference in survival with α = 0.05, β = 0.2) and intranasally administered with the indicated nanohydrogel formulations three times at 3-day intervals. Each dose contained 50 μg of OVA peptide in a total volume of 50 μL (25 μL per nostril), administered bilaterally via a micropipette. Mice were lightly anesthetized with isoflurane (induction: 3%, maintenance: 1.5%) during intranasal administration and placed in a supine position. For IVIS imaging, D-luciferin (150 mg/kg in PBS) was administered intraperitoneally 10 min prior to imaging. Images were acquired with an exposure time of 1–5 min. BLI was performed every 7 days during the first 21 days.

### 2.11. Activation of T Lymphocytes In Vitro

Seven days after final immunization, tumor-bearing mice were sacrificed by decapitation under deep anesthesia. Tumors were excised from mice and immediately placed in ice-cold RPMI-1640 medium supplemented with 1% penicillin-streptomycin. The tissues were minced into small fragments (approximately 1–2 mm^3^) using sterile surgical scissors. The minced tumor pieces were then transferred to a digestion cocktail containing RPMI-1640 with 1 mg/mL collagenase type IV, 0.2 mg/mL hyaluronidase, and 50 U/mL Dnase I, and incubated at 37 °C for 30–60 min with gentle shaking. After digestion, the cell suspension was passed through a 70 μm cell strainer to remove undigested debris and washed twice with phosphate-buffered saline (PBS) containing 2% fetal bovine serum (FBS). Red blood cells were lysed using ACK lysis buffer for 3–5 min at room temperature, and the lysis reaction was stopped by adding PBS with 2% FBS. The remaining cells were then washed and resuspended in complete RPMI-1640 medium. The single-cell suspension was ready for subsequent immune cell isolation via density gradient centrifugation and counted using a hemocytometer. The lymphocytes harvested from tumors were added to 96 well plate (5 × 10^4^ cells/well). OVA peptide (1 μg/mL) and IL-2 (10 ng/mL) were added to stimulate OVA-specific T cells. The cells were cultured for 72 h at 37 °C in a 5% CO_2_ incubator. Six hours prior to cell collection, brefeldin A was added to block intracellular cytokine secretion. After cell collection, FITC-CD3 and PE-CD8 antibodies were added and incubated at 4 °C for 15 min followed by treatment with fixation buffer for 20 min at 4 °C. The fixed cells were then washed with CytoPerm wash buffer and incubated with APC-conjugated anti-IFN-γ for 20 min at room temperature. After two additional washes with CytoPerm wash buffer, the cells were analyzed using a flow cytometer (BD Biosciences, San Jose, CA, USA). CCK-8 assay was employed to measure the proliferation levels of T lymphocytes in vitro.

### 2.12. Immunohistochemical Analysis and Immunofluorescence Staining

Tumor tissues and major organs (brain and olfactory bulb) were collected and post-fixed in 4% PFA for 24 h, followed by paraffin embedding. 4 μm sections were placed on glass slides. Tissue sections were deparaffinized in xylene and rehydrated through a graded series of ethanol (100%, 95%, 80%, and 70%) to distilled water. Antigen retrieval was performed by heating the sections in citrate buffer (10 mM, pH 6.0) or EDTA buffer (1 mM, pH 8.0) at 95–100 °C for 10–20 min, followed by cooling to room temperature. Endogenous peroxidase activity was blocked by incubating the sections in 3% H_2_O_2_ in methanol for 10–15 min at room temperature. After washing three times with phosphate-buffered saline (PBS), the sections were blocked with 5% normal goat serum (or 5% bovine serum albumin) in PBS containing 0.1% Triton X-100 for 1 h at room temperature to prevent non-specific binding. For immunohistochemical analysis, the sections were then incubated with anti-Ki67 antibodies diluted in blocking buffer overnight at 4 °C in a humidified chamber. The following day, the sections were washed three times with PBS and incubated with the appropriate horseradish peroxidase (HRP)-conjugated secondary antibody for 1 h at room temperature. After additional washes, the signal was visualized using 3,3′-diaminobenzidine (DAB) substrate, and the reaction was terminated by rinsing with distilled water. Finally, the sections were counterstained with hematoxylin, dehydrated through graded ethanol, cleared in xylene, and mounted with coverslips using a permanent mounting medium. Images were captured under a light microscope. For immunofluorescence staining, the rehydrated sections were exposed to fluorescein-labeled anti-CD4, CD8, F4/80, CD11b, NK1.1, CD11c, and GFAP antibodies overnight at 4 °C and then washed with PBS containing 0.05% Tween 20 (PBST). DAPI staining was subsequently performed in the dark. Finally, fluorescence images were acquired by microscope slide scanner (3DHISTECH Pannoramic SCAN II, Budapest, Hungary). Antibody information is shown in Table S2.

### 2.13. Biological Safety Analysis

For cytotoxicity evaluation in vitro, 293T cells (obtained from American Type Culture Collection, ATCC) were seeded in 96-well plates at a density of 1 × 10^4^ cells/well in DMEM containing 10% FBS. After 24 h of attachment, the cells were exposed to nanoCOM-GEL-OVA at escalating concentrations (1, 3, 10, 30 mg/mL) for 24 h. Cell viability was measured using the CCK-8 assay (Beyotime Biotec, Shanghai, China) according to the manufacturer’s protocol. Each concentration was tested in triplicate.

To assess the safety of nanoCOM-GEL-OVA in vivo, normal C57BL/6 mice (female, 6–8 weeks) were randomly assigned to two groups (*n* = 4 per group): PBS (control) and nanoCOM-GEL-OVA (50 μL, 1 mg/mL). All formulations were intranasally administered once every five days over a 15-day period (total of 3 doses). Body weight was measured every three days. For serum biochemical analysis, mice were euthanized by CO_2_ asphyxiation followed by blood collection via retro-orbital puncture into EDTA-coated tubes and tissue collection. The plasma was separated by centrifugation (3000 rpm, 10 min, 4 °C). Serum levels of ALT, AST, UREA, and CREA were measured using commercially available kits (Nanjing Jiancheng Bioengineering Institute, Nanjing, China) according to the manufacturer’s protocols. Cervical dislocation was performed on day 2 after the final administration.

### 2.14. Statistical Analysis

Data are presented as mean ± standard error of the mean (S.E.M.) throughout the manuscript. Statistical comparisons among multiple groups were performed using one-way analysis of variance (ANOVA) followed by Tukey’s post hoc test for multiple comparisons. Survival curves were analyzed using the Kaplan–Meier method with the log-rank (Mantel–Cox) test. All experiments were performed with at least three biological replicates, and sample sizes (*n*) for each experiment are indicated in the corresponding figure legends. Sample size was determined by a prospective power analysis using G*Power software (version 3.1). Based on our preliminary data, we assumed a 30% difference in the median survival between treatment groups, with a standard deviation of 25%. With a two-sided log-rank test, α = 0.05, and β = 0.2 (power = 80%), the required sample size was calculated to be 7 animals per group. To account for a potential 10% attrition rate, we enrolled 8 animals per group. Flow cytometry data were analyzed with FlowJo VX software (BD Biosciences). All statistical analyses were conducted using GraphPad Prism 8.0 (GraphPad Software). A *p* value of less than 0.05 was considered statistically significant (* *p* < 0.05, ** *p* < 0.01, *** *p* < 0.001, **** *p* < 0.0001).

## 3. Results

### 3.1. Characteristics of NanoCOM-GEL Vaccine

**Figure 1:**
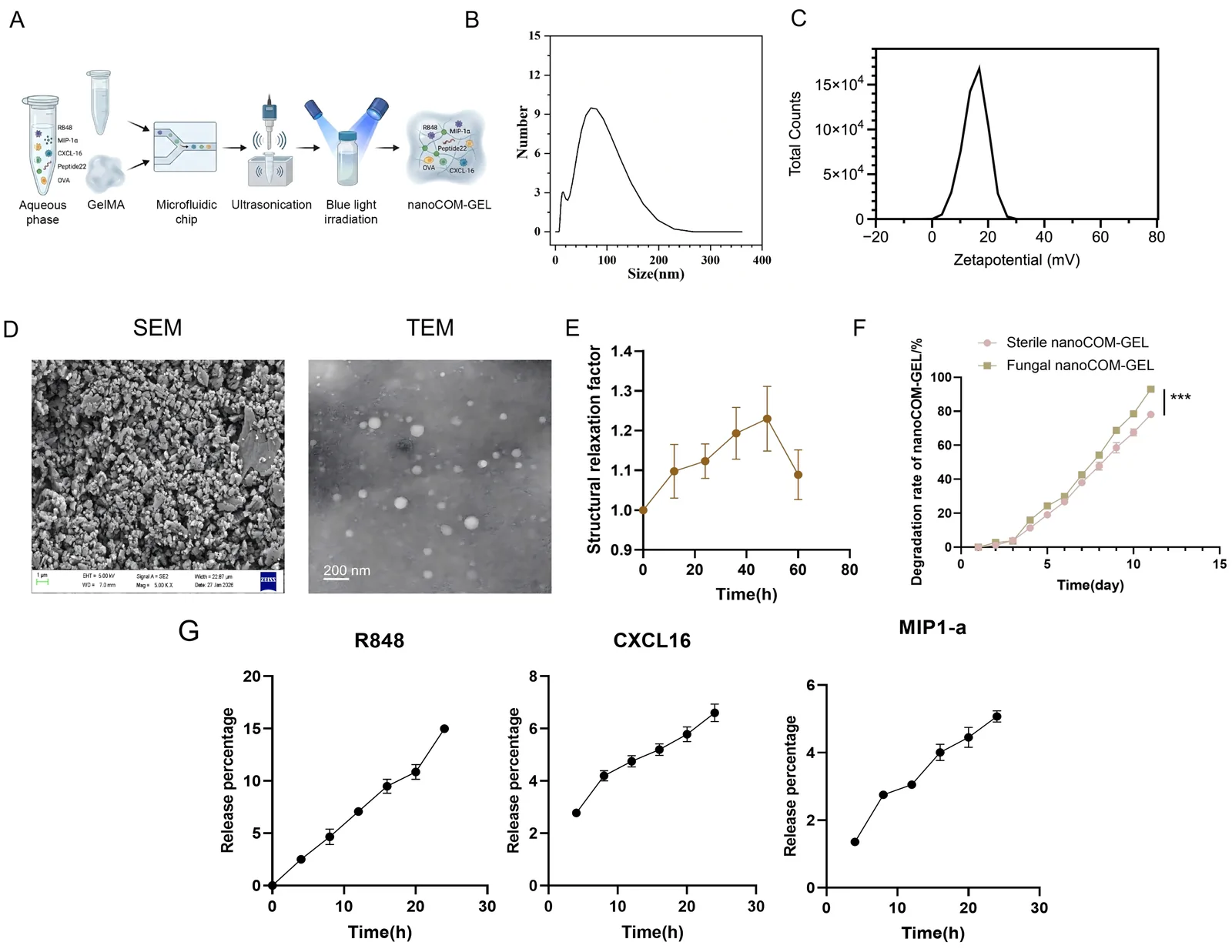
Characteristics of nanoCOM-GEL vaccine. (**A**) Schematic diagram of synthesis of nanoCOM-GEL vaccine; (**B**) the particle size distribution of nanoCOM-GEL; (**C**) zeta potential of nanoCOM-GEL; (**D**) SEM and TEM images of nanoCOM-GEL; (**E**) dimensional stability factor variation curve of nanoCOM-GEL. Data are expressed as mean ± S.E.M, *n* = 3; (**F**) degradation rates of nanoCOM-GEL in bacterial and sterile environments in vitro. Data are expressed as mean ± S.E.M, *n* = 3. One-way ANOVA followed by Tukey’s multiple comparisons test was applied after confirming the assumptions of normality (Shapiro–Wilk test) and homogeneity of variance (Brown–Forsythe test). *** *p* < 0.001; (**G**) time-dependent release profiles of CXCL16, MIP-1α, and R848 from nanoCOM-GEL in vitro. Data are expressed as mean ± S.E.M, *n* = 3.

Firstly, a nanocomposite hydrogel (nanoCOM-GEL) vaccine was constructed by loading pep22 peptide, R848, MIP-1α, and CXCL16 protein into nanoGelMA hydrogel (Figure 1A). As shown in Figure 1B, nanoCOM-GEL exhibited a narrow particle size distribution profile, with an average diameter of approximately 101 nm. Its zeta potential was about +20 mV (Figure 1C). The scanning electron microscopy (SEM) images clearly showed that the particles were regularly spherical or spheroidal, with a porous structure on their surface. This porous structure is crucial for high loading capacity of peptide antigens and subsequent controlled release. The transmission electron microscope (TEM) images further confirm its nanoscale hydrogel (Figure 1D).

For hydrophobic nanogels, dimensional stability is intrinsically coupled with drug retention capacity [21]. To characterize the structural stability of the hydrophobic nanogel, we measured its volume change from its most desiccated state in an aqueous environment. The material exhibited a dimensional stability factor of 1.1–1.2 (Figure 1E). The nanohydrogel exhibited excellent dimensional stability, with only a ~10–20% volume change, consistent with the limited swelling behavior typical of nanoscale hydrogel systems. The intrinsic dimensional stability of the nanohydrogel is a desirable feature for a sustained-release vaccine depot and a critical consideration for intranasal administration to avoid nasal obstruction [22].

For a peptide vaccine delivery system, the characteristics of its encapsulation efficiency, degradation, and controlled release are important for its efficacy and safety. The encapsulation efficiencies of OVA peptide, R848, MIP-1α, and CXCL16 in the nanoCOM-GEL formulation ranged from 60% to 80%, indicating good encapsulation efficacy (Figure S1). Next, its degradation kinetics were examined in vitro. As illustrated in Figure 1F, the vaccine displayed only an 80% degradation rate up to 10 days under sterile or non-sterile conditions, demonstrating favorable biodegradability. After 10 days, in a non-sterile setting, the degradation rate of the nanoCOM-GEL vaccine was higher than that under aseptic conditions. Furthermore, the nanoCOM-GEL vaccine also showed sustained-release profiles for both CXCL16 and MIP-1α. Over the 24 h period, the cumulative release of CXCL16 reached approximately 6%, whereas that of MIP-1α was 4.5%. By contrast, R848 showed more uniform release behavior, with a considerably faster release rate than CXCL16 and MIP-1α, achieving a cumulative release of about 15% within 24 h (Figure 1G).

Due to diffusion, hydrogel degradation and dimensional stability, the controlled release characteristics of nanoCOM-GEL ensured long-term antigen retention at the tumor site or within draining lymph nodes [23]. This provides enough time for capture and the processing of the antigen by antigen-presenting cells (APCs, such as dendritic cells), which help to break immune tolerance and induce durable immunological memory. Moreover, the controllable degradation rate and sustained release profile are beneficial for adaptive immune response initiation and expansion because they can enhance DC maturation, migration of immune cells to lymph nodes, and activation of naive T cells.

### 3.2. The Effect of NanoCOM-GEL Vaccine on Attraction and Activation of Immune Cells In Vitro

**Figure 2:**
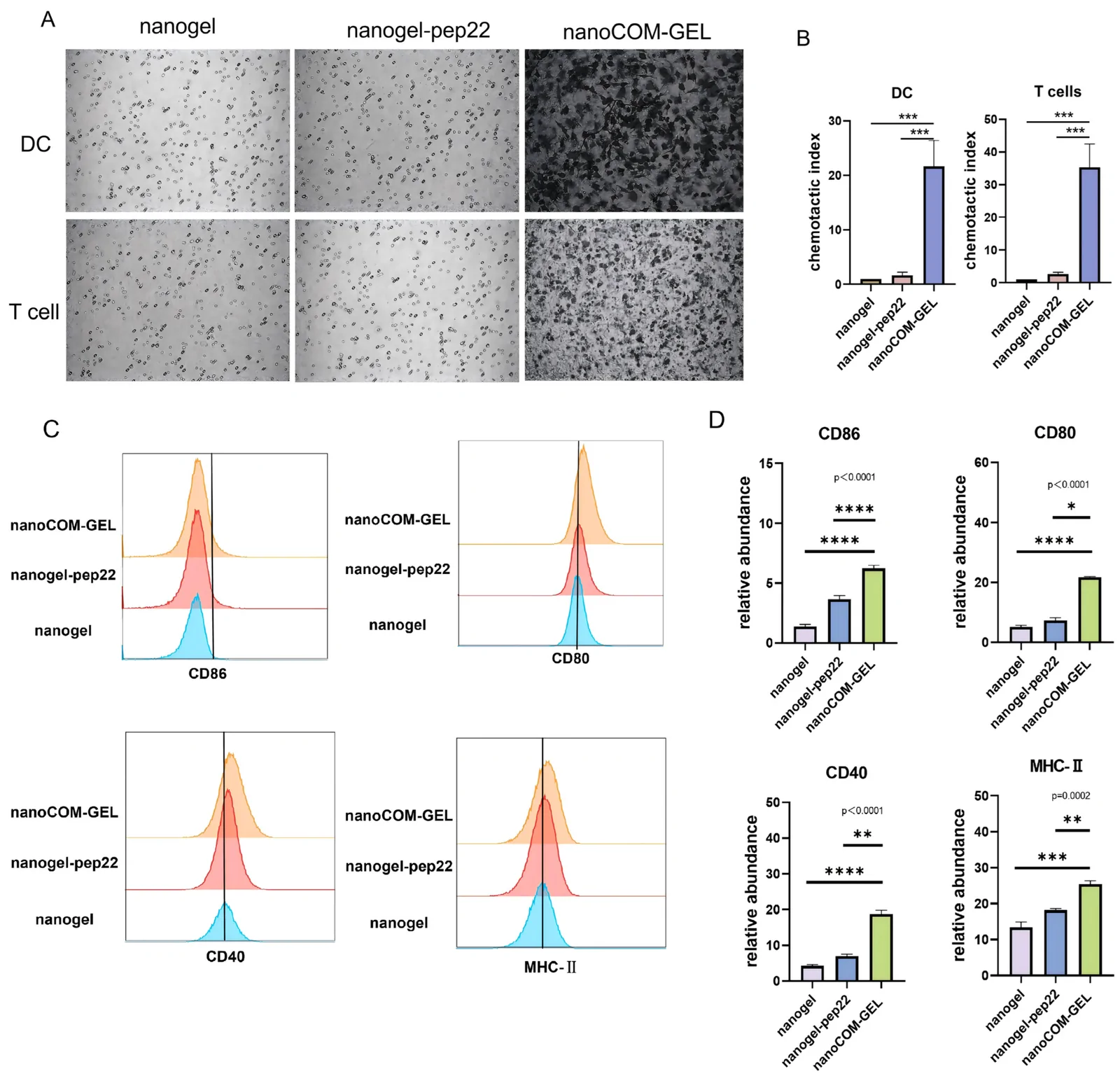
Effects of nanoCOM-GEL vaccine on attraction and activation of immune cells in vitro. (**A**,**B**) Representative light microscopy images of DC and T cell migration induced by various nanohydrogels as indicated in figures (200× magnification) and quantitative analysis. (**C**,**D**) Representative flow cytometry images showing expression of co-stimulatory molecules on DCs after treatment with various nanohydrogels as indicated in figures and quantitative analysis. All data are expressed as mean ± S.E.M., *n* = 4. One-way ANOVA followed by Tukey’s multiple comparisons test was applied after confirming the assumptions of normality (Shapiro–Wilk test) and homogeneity of variance (Brown–Forsythe test). * *p* < 0.05; ** *p* < 0.01; *** *p* < 0.001; **** *p* < 0.0001.

Recruitment and activation of immune cells within the tumor are important for the initiation of anti-tumor immunity [24]. Therefore, a transwell assay was used to assess the ability of the nanoCOM-GEL vaccine to attract dendritic cells (DCs) and T cells in vitro. Due to the chemotactic effects of CXCL16 and MIP-1α, the nanoCOM-GEL induced the strongest migration of DCs and T cells. Specifically, DC migration increased to almost eight times that of the blank nanogel group, while T cell migration rose to approximately 36 times that of the blank nanogel group (Figure 2A,B). These findings suggested that the chemokines within the nanoCOM-GEL vaccine potently enhanced the migration of both DCs and T cells.

The maturation of dendritic cells (DCs) is also important for initiation of adaptive immune responses. Matured DCs upregulate antigen-presenting molecules (MHC-II) and co-stimulatory molecules (such as CD40, CD80, CD86), which are crucial for effectively activating naive T cells [25]. Therefore, we next examined the capacity of the nanoCOM-GEL to induce DC maturation in vitro. Among all tested groups, the nanoCOM-GEL vaccine induced the highest levels of co-stimulatory molecule expression, indicating the strongest ability to promote DC maturation (Figure 2C,D). These results confirmed that the nanoCOM-GEL effectively enhanced the maturation of DCs. Furthermore, previous studies showed that macrophages (TAM) were the most abundant immune cell population in the tumor microenvironment (TME), accounting for 30–50% of infiltrating immune cells in GBM [26]. Activated M1-type macrophages exert antitumor effects by secreting pro-inflammatory mediators such as TNF-α, IL-12, and ROS. In contrast, M2-type macrophages promote angiogenesis, matrix remodeling, T cell suppression and tumor progression [27]. To investigate whether nanoCOM-GEL can induce TAM polarization toward the M1 phenotype, we co-incubated different nanohydrogel vaccines with primary mouse macrophages and examined the expression of M1/M2-related cytokines by RT-PCR(The primers for cytokine genes were listed in Table S3). The results showed that nanoCOM-GEL promoted the upregulation of M1-type cytokines (IL-12, TNF-α, iNOS) in macrophages (Figure S2). These findings suggested that nanoCOM-GEL could skew TAM polarization to M1, which could reshape the immunosuppressive tumor microenvironment.

### 3.3. The Effect of NanoCOM-GEL Vaccine on Crossing of BBTB Model and Targeting the GBM In Vitro

**Figure 3:**
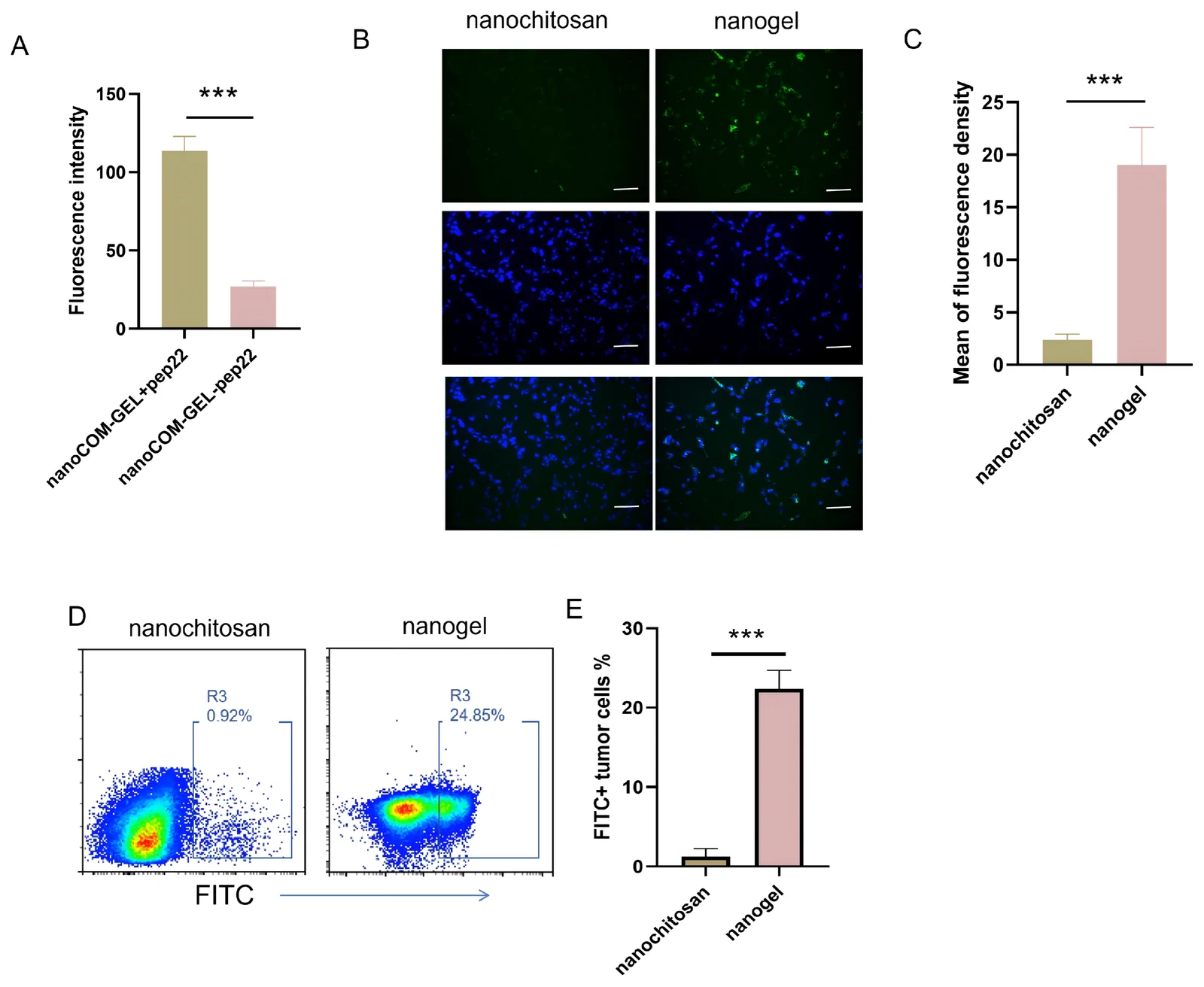
The ability of the nanoCOM-GEL vaccine to transfer across the BBTB model and targeting the GBM in vitro. (**A**) Quantitative analysis of fluorescence intensity (FITC) illustrating the ability of nanoCOM-GEL containing or lacking peptide 22 to transfer across the BBB model in vitro. (**B**,**C**) Fluorescence microscope images showing the ability of nanogel synthesized with GelMA in binding glioblastoma cell line and quantitative analysis. Scale bar = 50 μm. (**D**,**E**) Representative flow cytometry images showing the ability of nanogel synthesized with GelMA in binding glioblastoma cell line and quantitative analysis. All data are expressed as mean ± S.E.M, *n* = 3. One-way ANOVA followed by Tukey’s multiple comparisons test was applied after confirming the assumptions of normality (Shapiro–Wilk test) and homogeneity of variance (Brown–Forsythe test). ***, *p* < 0.001.

The ability of a vaccine to traverse the blood–brain tumor barrier (BBTB) and reach GBM cells after entering the brain can elicit a potent intratumoral immune response [28]. Given that the ability of peptide 22 to transfer across the BBTB via binding to specific receptors on BBTB endothelial cells [17], we next investigated whether peptide 22 enhanced the ability of the nanoCOM-GEL in a model of the BBTB in vitro. The FITC labeled nanoCOM-GEL or nanoCOM-GEL without peptide 22 were put in the model of the BBTB for 1 h, respectively. As shown in Figure 3A, a fluorescence spectrophotometer revealed that nanoCOM-GEL transferred across the BBTB model more efficiently compared with nanoCOM-GEL without peptide 22. The fluorescence intensity from the nanoCOM-GEL group is fivefold higher than that of the nanoCOM-GEL without peptide 22 group. The findings suggested that the peptide 22 substantially improved the ability of nanoCOM-GELs to cross the BBTB and potentially facilitated entry into tumors. Moreover, the small particle size of nanoCOM-GELs enabled them to penetrate local capillaries, as evidenced by the fact that nanoCOM-GEL without peptide 22 could cross the BBTB moderately.

Another ability of this vaccine to target the GBM lesion and accumulate within the tumor could attract immune cells into the tumor and is also important for intratumoral immunity. Given that the RGD motif in GelMA can bind to the integrin αvβ3 on the tumor surface [29,30], we next verified whether nanoCOM-GEL could target GBM. FITC-labeled nanoCOM-GEL or nanochitosan made from chitosan was incubated with tumor cells. After thorough washing, the binding ability of nanoCOM-GEL to the tumor cells was observed using immunofluorescence and flow cytometry. As shown in Figure 3B,C, the fluorescent signals of the nanoCOM-GEL group were stronger than those of the nanochitosan group. Flow cytometry results also showed that when incubated with nanoCOM-GEL, the percentage of FITC+ tumor cells reached 25%, which was significantly higher than that in the nanochitosan control group (Figure 3D,E). To confirm that the nanogel targets glioma cells through the binding of GelMA to integrin αvβ3, we have now performed the blocking experiments using a neutralizing anti-αvβ3 integrin antibody (2 μg/mL) administered 2 h prior to nanoCOM-GEL administration. The result showed that antibody blockade significantly reduced tumor accumulation of the fluorescently labeled nanohydrogel, with a ~92% decrease in fluorescence signal compared to the isotype control group (Figure S3). The results suggested that nanoCOM-GEL could bind to glioma cells and possess tumor-targeting properties via the GelMA component.

### 3.4. Intranasal Administration of NanoCOM-GEL Results in an Effective Accumulation in the Brain

**Figure 4:**
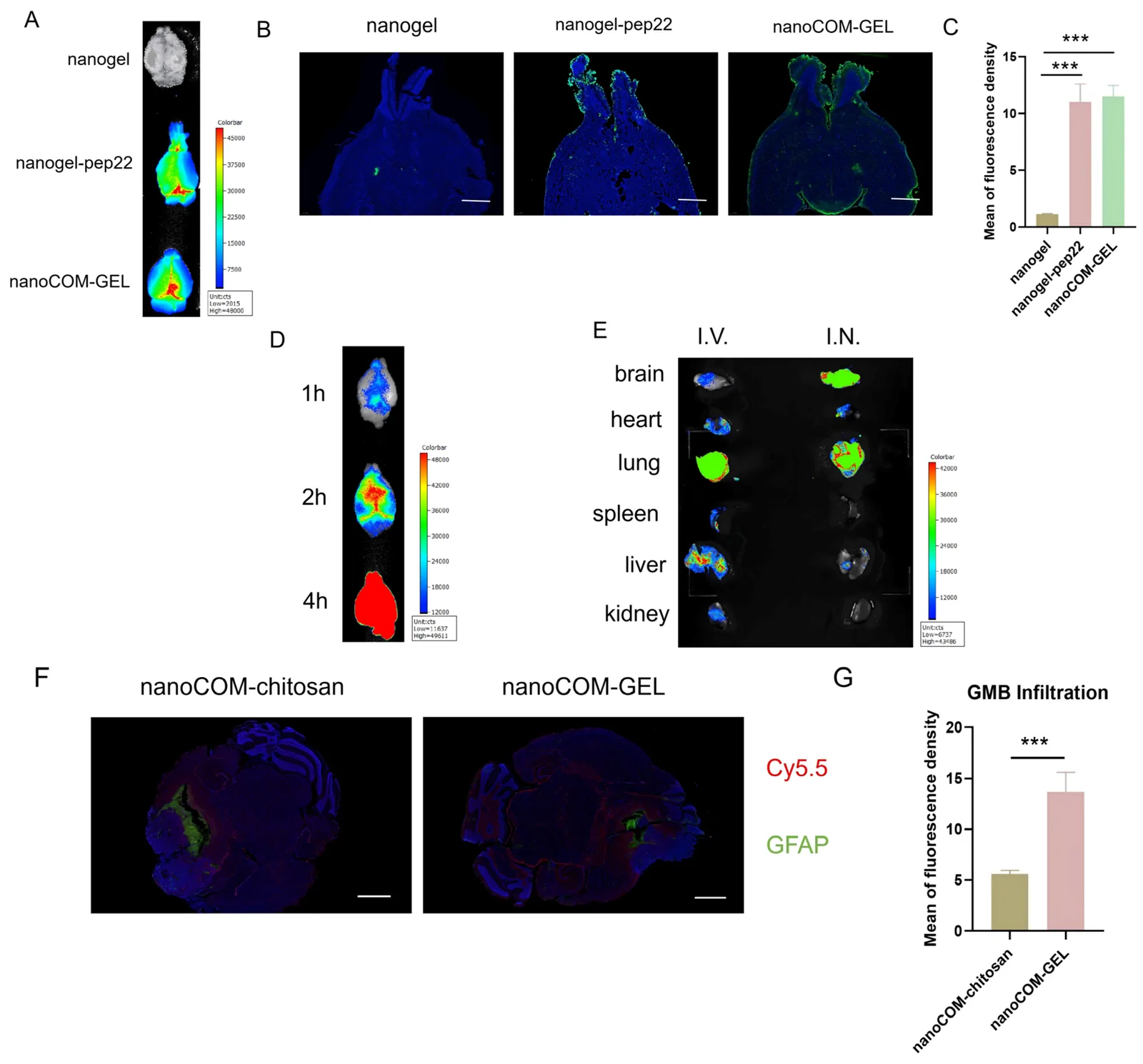
The ability of the nanoCOM-GEL vaccine to bypass the BBB and target GBM after intranasal administration in vivo. (**A**) Using the IVIS imaging system, brain distribution of Cy2-labeled nanogel, nanogel-pep22, and nanoCOM-GEL was assessed at 2 h post-administration. (**B**,**C**) Fluorescence microscopy was used to detect brain localization of Cy2-labeled nanohydrogels 2 h after intranasal administration and quantitative analysis. Scale bar = 500 μm. (**D**) Time-dependent brain accumulation of Cy2-labeled nanoCOM-GEL was monitored by IVIS at 1, 2, and 4 h after intranasal administration ex vivo. (**E**) A comparison of Cy2-labeled nanoCOM-GEL distribution was observed via IVIS at 2 h post-administration using two different routes: intranasal (I.N.) versus intravenous (I.V.) injection. (**F**,**G**) In GBM-bearing mice, brain distribution of Cy5.5-labeled nanoCOM-GEL and nanoCOM-chitosan was examined 10 h after intranasal administration, accompanied by quantitative analysis. All results are expressed as mean ± S.E.M. (*n* = 3). One-way ANOVA followed by Tukey’s multiple comparisons test was applied after confirming the assumptions of normality (Shapiro–Wilk test) and homogeneity of variance (Brown–Forsythe test). ***, *p* < 0.001; Scale bar = 500 μm.

Based on in vitro experiments showing that nanoCOM-GEL could effectively transfer across the BBB model and target tumor cells, we first investigated whether intranasal administration of nanoCOM-GEL could bypass the BBB in vivo. The nanoCOM-GEL labeled with Cy2 was intranasally given to mice. Then, the brain was collected and the distribution of nanoCOM-GEL in brains was detected using an in vivo imaging system (IVIS) ex vivo. In the brains of treated mice, the Cy2 signal intensity of nanoCOM-GEL and nanogel-pep22 was significantly higher than that of nanogel without peptide 22, indicating the importance of peptide 22 in enhancing the retention of nanoCOM-GEL in the brain parenchyma (Figure 4A–C). In addition, we observed the dynamic infiltration process of nanoCOM-GEL in the brain. The accumulation in the brain peaked at 4 h after intranasal administration of nanoCOM-GEL (Figure 4D). To verify that intranasal administration of nanoCOM-GEL bypasses the BBB more efficiently compared to other routes of administration, the differences between intranasal and intravenous delivery were assessed after administration of Cy2-labeled nanoCOM-GEL. As shown in Figure 4E, nanoCOM-GEL tends to accumulate in the brain after intranasal administration, while intravenous injection leads to predominant accumulation in the liver. This is evidenced by a much higher Cy2 signal in the brain and significantly lower accumulation in the liver in mice treated intranasally with nanoCOM-GEL compared to those treated intravenously with nanoCOM-GEL.

Next, to further elucidate whether the GelMA component enabled nanoCOM-GEL to specifically accumulate in GBM tumors, brain tissue was collected from the mice to detect nanoCOM-GEL distribution within the brain by immunofluorescence after intranasal administration of Cy5.5-labeled nanoCOM-GEL in tumor-bearing mice. As shown in Figure 4F,G, higher fluorescence intensity Cy5.5 signals colocalized with glial fibrillary acidic protein (GFAP) was observed in the brain from the nanoCOM-GEL group compared with that of the nanoCOM-chitosan group, which indicated that nanoCOM-GEL could accumulate within the tumor due to the RGD motif in the GelMA component. Accumulation of nanoCOM-GEL in the tumor provides a route for intratumoral immunity. Previous studies have shown that intratumoral immunity directly remodels the tumor microenvironment and induces immunogenic cell death, thereby efficiently activating tumor-specific T cells [31,32]. This strategy lays the foundation for achieving intratumoral immunity against GBM via intranasal immunization.

### 3.5. Intranasal Administration of NanoCOM-GEL-OVA Significantly Suppressed the Growth of GBM

**Figure 5:**
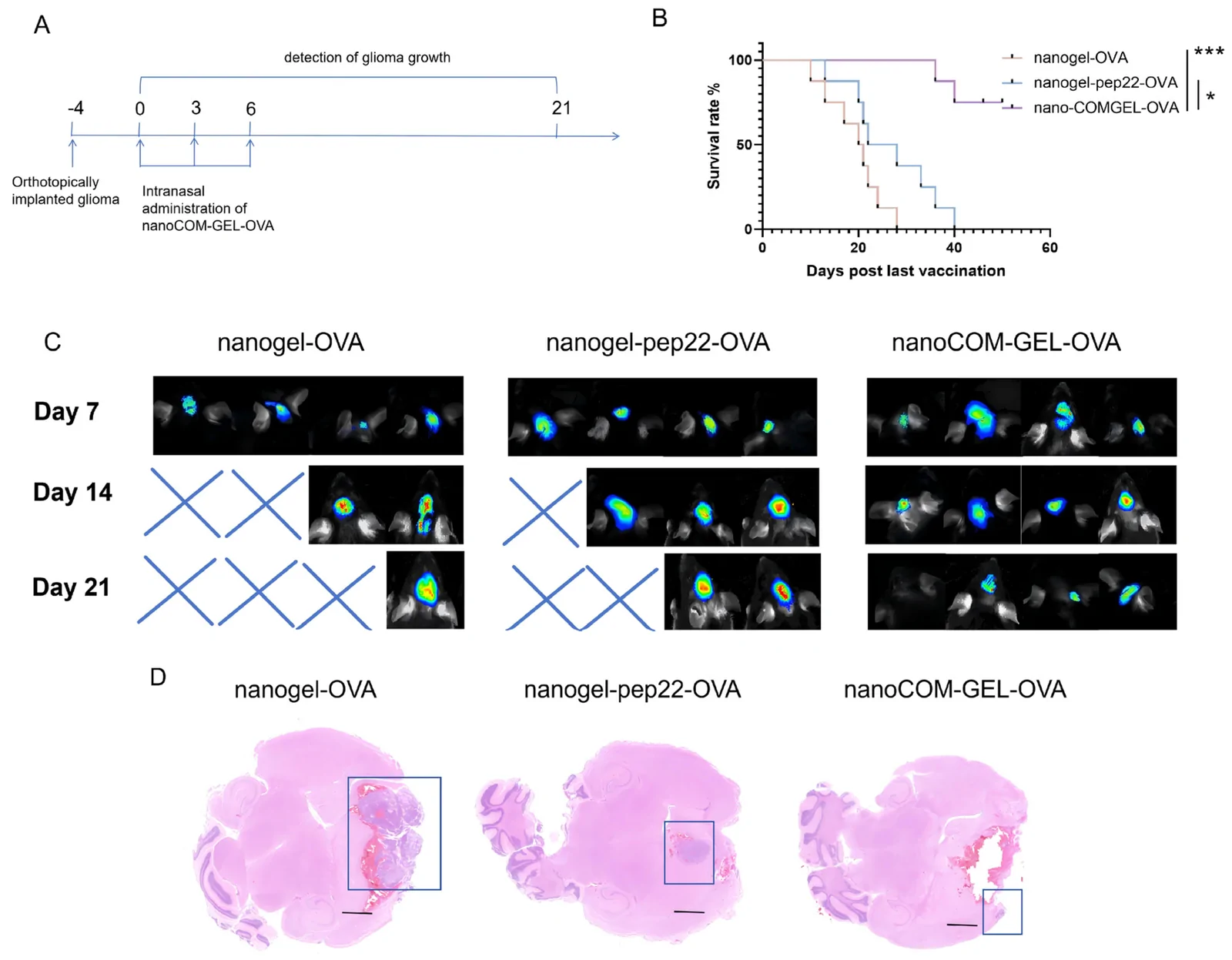

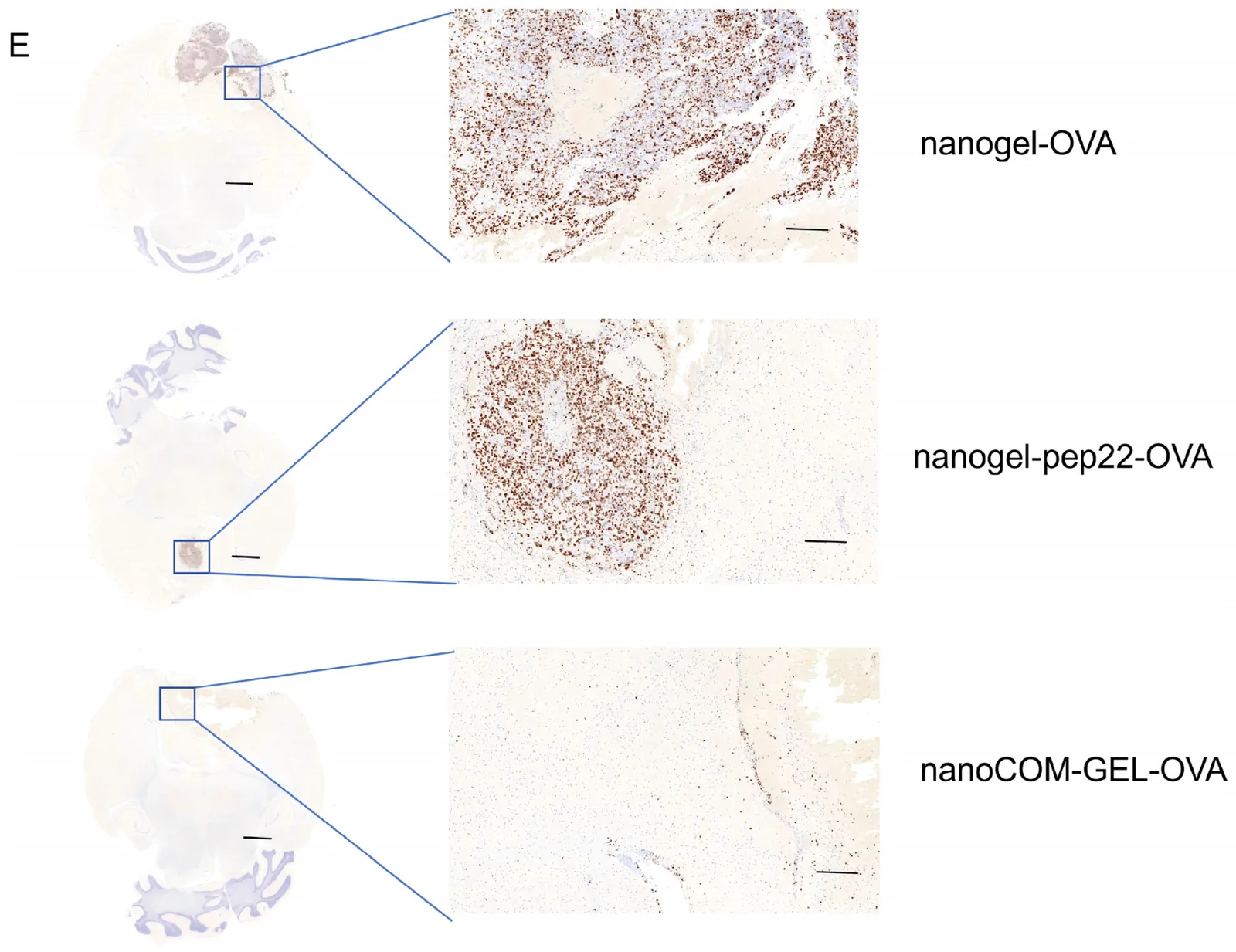
The therapeutic effects of nanoCOM-GEL-OVA vaccine. (**A**) A schematic timeline illustrating the immunization regimen with nanoCOM-GEL-OVA and the subsequent experimental design for evaluating anti-GBM effects. (**B**) Survival analysis of mice receiving intranasal immunization with different nanohydrogel formulations is presented as Kaplan–Meier curves. * *p* < 0.05, *** *p* < 0.001. Statistical significance was determined using log-rank (Mantel–Cox) test (*n* = 8 per group). (**C**) Longitudinal bioluminescence imaging captured at multiple time points from mice intranasally immunized with the indicated nanohydrogel formulations. (**D**) Representative histology images showing H&E-stained brain sections from each treatment group. The boxed regions in the histological images (Panel **D**) indicate tumor tissue. Scale bars: 500 μm. (**E**) Representative histology images showing immunohistochemical staining against Ki-67 in brain sections from each treatment group. Scale bars: 500 μm (**left**) and 50 μm (**right**).

The therapeutic effect of nanoCOM-GEL was evaluated in an orthotopic GL261-OVA-luc GBM mouse model. The treatment schedule was outlined in Figure 5A. As shown in Figure 5B, mice intranasally immunized with nanoCOM-GEL-OVA exhibited substantial tumor suppression, achieving a complete remission (CR) rate of 50% (4 out of 8 animals) over a 60-day observation period. While the antitumor effect of nanogel-pep22-OVA was relatively modest, no CRs were found until 40 days after immunization. GBM progression was also monitored using the IVIS imaging system (Figure 5C). In the nanogel-OVA group, tumor growth was visible by IVIS as early as 7 days post-inoculation and the mice died shortly thereafter. Kaplan–Meier survival analysis revealed that the median survival time was 20.5 days for the nanogel-OVA group, 25.0 days for the nanogel-pep22-OVA group and was not reached (>55 days) for the nanoCOM-GEL-OVA group. Cox regression analysis demonstrated that nanoCOMGEL-OVA significantly reduced mortality risk compared with both nanogel-OVA (HR = 0.09, 95% CI: 0.020.35, *p* < 0.001) and nanogel-pep22-OVA (HR = 0.20, 95% CI: 0.050.75, *p* = 0.017). No significant difference was observed between nanogel-pep22-OVA and nanogel-OVA (HR = 0.44, 95% CI: 0.161.19, *p* = 0.104).

To further confirm the therapeutic effect of nanoCOM-GEL-OVA, brain tissues were harvested on day 21 after tumor implantation and processed for H&E and immunohistochemical staining. As illustrated in Figure 5D, the largest tumor area was found in the brain sections from nanogel-OVA-treated mice, whereas those from the nanogel-pep22-OVA group showed comparatively smaller lesions. Strikingly, almost complete tumor eradication was observed in the nanoCOM-GEL-OVA-treated mice, with no evidence of GL261-OVA-luc cell proliferation in the right hemisphere. Moreover, in contrast to the other treatment groups, histological analysis also showed the absence of Ki-67-positive cells in brain tissues from the nanoCOM-GEL-OVA group, indicating markedly reduced GBM cell proliferation (Figure 5E). Together, these findings demonstrated that nanoCOM-GEL-OVA induced potent anti-GBM immunity and had excellent therapeutic effects.

### 3.6. Intranasal Administration of NanoCOM-GEL-OVA Elicited Robust Anti-Tumor Immunity

**Figure 6:**
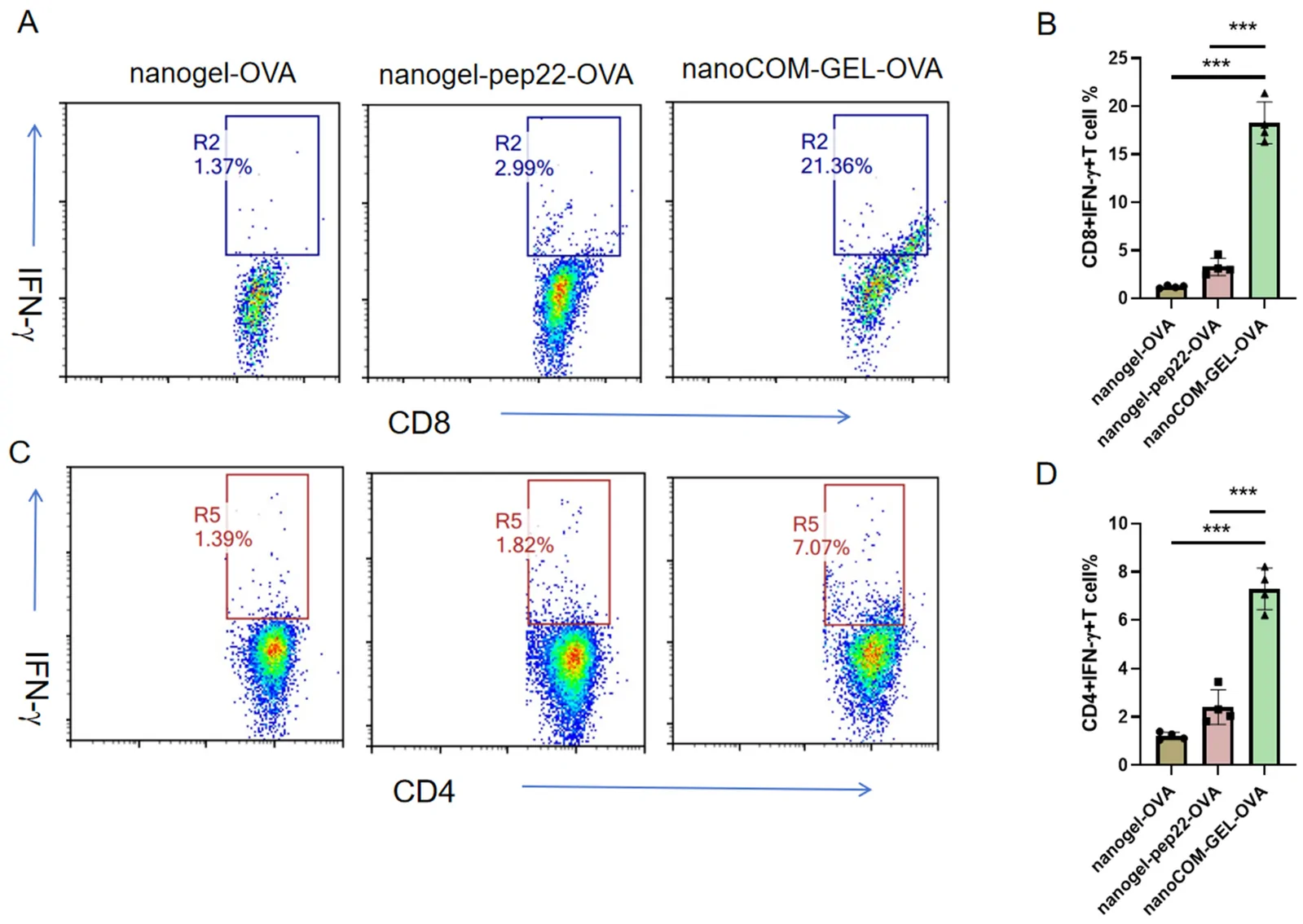

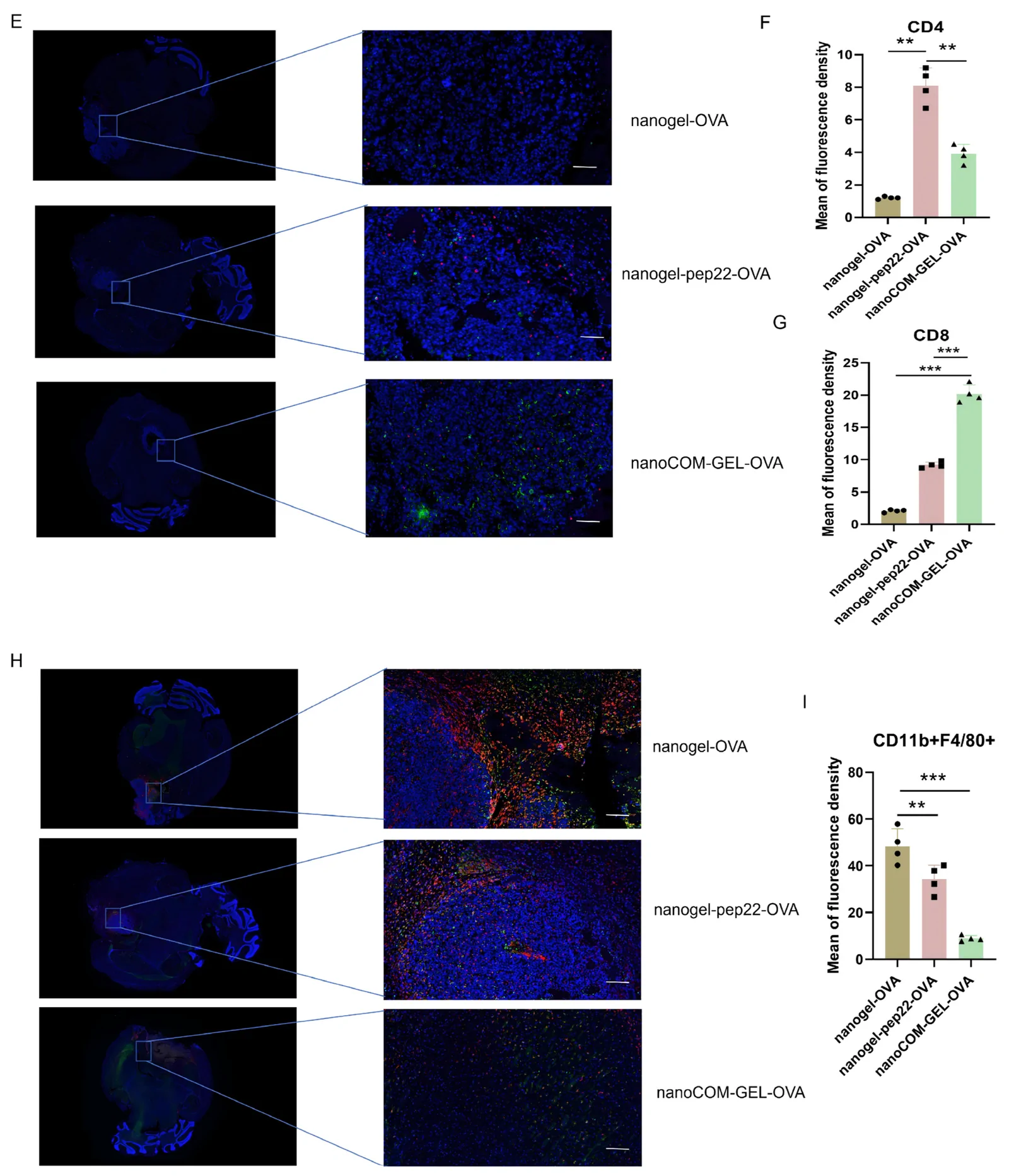
The effects of nanoCOM-GEL-OVA vaccines on anti-tumor immunity. (**A**–**D**) Scatter plots showing the frequencies and quantitative analysis of CD8^+^ IFN-γ^+^ T cells (**A**,**B**) and CD4^+^ IFN-γ^+^ T cells (**C**,**D**) in brain of mice intranasally immunized with the indicated nanohydrogel formulations. (**E**) The images of immunofluorescence staining of brain slices after mice were intranasally administrated various nanohydrogels, as indicated in the figure. Green: CD8; red: CD4; blue: nuclei stained by DAPI. Scale bars: 50 μm. (**F**,**G**) Quantitative analysis of the expression of CD4^+^ (F), CD8^+^ (**G**) T cells in the brain after different treatments (*n* = 4). (**H**,**I**) The images of immunofluorescence staining of brain slices after mice were intranasally administrated various nanohydrogels, as indicated in the figure and quantitative analysis. Red: F4−80; green: CD11b; blue: nucleus stained by DAPI. Scale bars: 50 μm. Data are expressed as mean ± S.E.M. (*n* = 4). One-way ANOVA followed by Tukey’s multiple comparisons test was applied after confirming the assumptions of normality (Shapiro–Wilk test) and homogeneity of variance (Brown–Forsythe test). ** *p* < 0.01, *** *p* < 0.001.

To verify whether intranasal administration of the nanoCOM-GEL-OVA vaccine can trigger an intratumoral or systemic antigen-specific immune response, we separated T cells from tumors after the final immunization and stimulated them in vitro with OVA protein. As shown in Figure 6A–D, the nanoCOM-GEL-OVA vaccine induced the highest percentage of CD8^+^ IFN-γ^+^ T cells and CD4^+^ IFN-γ^+^ T cells in total CD8^+^ T cells and total CD4^+^ T cells among all groups, reaching 21.36% in CD8^+^ T cells and 7.07% in CD4^+^ T cells, respectively. Moreover, compared to the other groups, the proliferation of T cells in tumors from the nanoCOM-GEL-OVA-immunized group significantly increased, reaching nearly 3.5 times and 1.5 times that of the control group, respectively (Figure S4A). These results indicated that intranasal immunization with nanoCOM-GEL-OVA vaccine could also trigger a significant intratumoral antigen-specific anti-tumor immunity.

Meanwhile, to evaluate whether intranasal immunization can elicit systemic antigen-specific immunity, we isolated T cells from the spleen and lymph nodes of immunized mice and assessed their proliferative response. As shown in Figure S4B,C, compared to the nanogel-OVA and nanogel-pep22-OVA groups, intranasal immunization with nanoCOM-GEL-OVA induced the proliferation of OVA-specific T cells derived from both the spleen and lymph nodes. These findings suggested that intranasal administration of nanoCOM-GEL-OVA not only elicited intratumoral (or intracerebral) antigen-specific immune responses but also induced systemic antigen-specific immune responses.

To achieve optimal therapeutic outcomes, tumor vaccines must effectively present tumor-associated antigens while counteracting the immunosuppressive tumor microenvironment [33]. Previous works demonstrated that VLP-based tumor vaccines effectively presented antigens to APCs and remodeled the TME by repolarizing M2-type macrophages [34]. Our data showed that nanoCOM-GEL-OVA significantly increased the fluorescence intensity of CD8, CD4 and CD11c signals compared to other groups (Figure 6E–G and Figure S5), indicating its key role in reshaping the TME and promoting antigen-specific T cell response and DC infiltration. Meanwhile, as shown in Figure 6H,I, fewer macrophages (CD11b+F4/80+) infiltrated the brains after treatment with the nanoCOM-GEL-OVA vaccine compared to the nanogel-OVA and nanogel-pep22-OVA groups. Intratumoral macrophages, known as tumor-associated macrophages (TAMs), are primarily of the M2 type, which can promote tumor growth and are the main contributors to the immunosuppressive microenvironment. NanoCOM-GEL-OVA reduced macrophage infiltration and alleviated their inhibitory effect on T cells to some extent. This suggested that nanoCOM-GEL-OVA could reshape the immunosuppressive tumor microenvironment. Furthermore, nanoCOM-GEL-OVA treatment could inhibit GBM progression and maintain BBB integrity, which avoided peripheral macrophage accumulation in tumors.

Meanwhile, we isolated intratumoral TAMs and examined the expression of M1/M2-related genes by RT-PCR. The results showed that in the nanogel-OVA and the nanogel-pep22-OVA immunization groups, the expression levels of M2-related genes such as TGF-β, IL-10, and CD206 in TAMs were significantly higher than those in the nanoCOM-GEL-OVA immunization group, while the expression of M1-related genes was markedly downregulated compared to the nanoCOM-GEL-OVA immunization group (Figure S6A). To confirm the polarization shift at the protein level, we performed immunofluorescence staining for iNOS (M1) and CD206 (M2) on tumor sections and detected serum cytokines. As shown in Figure S6B, nanoCOM-GEL-OVA significantly increased the fluorescence intensity of iNOS (M1) and decreased CD206 (M2) signal compared to other groups. Compared with the control group, the serum levels of M1-type cytokines (IFN-γ, IL-12, and TNF-α) were significantly elevated, indicating a systemic Th1-biased immune response induced by the vaccine (Figure S7).

Collectively, these results suggest that nanoCOM-GEL-OVA successfully remodels the immunosuppressive TME by activating T cells and decreasing TAMs, thereby eliciting robust anti-tumor immunity.

### 3.7. Biological Safety of NanoCOM-GEL Vaccine

**Figure 7:**
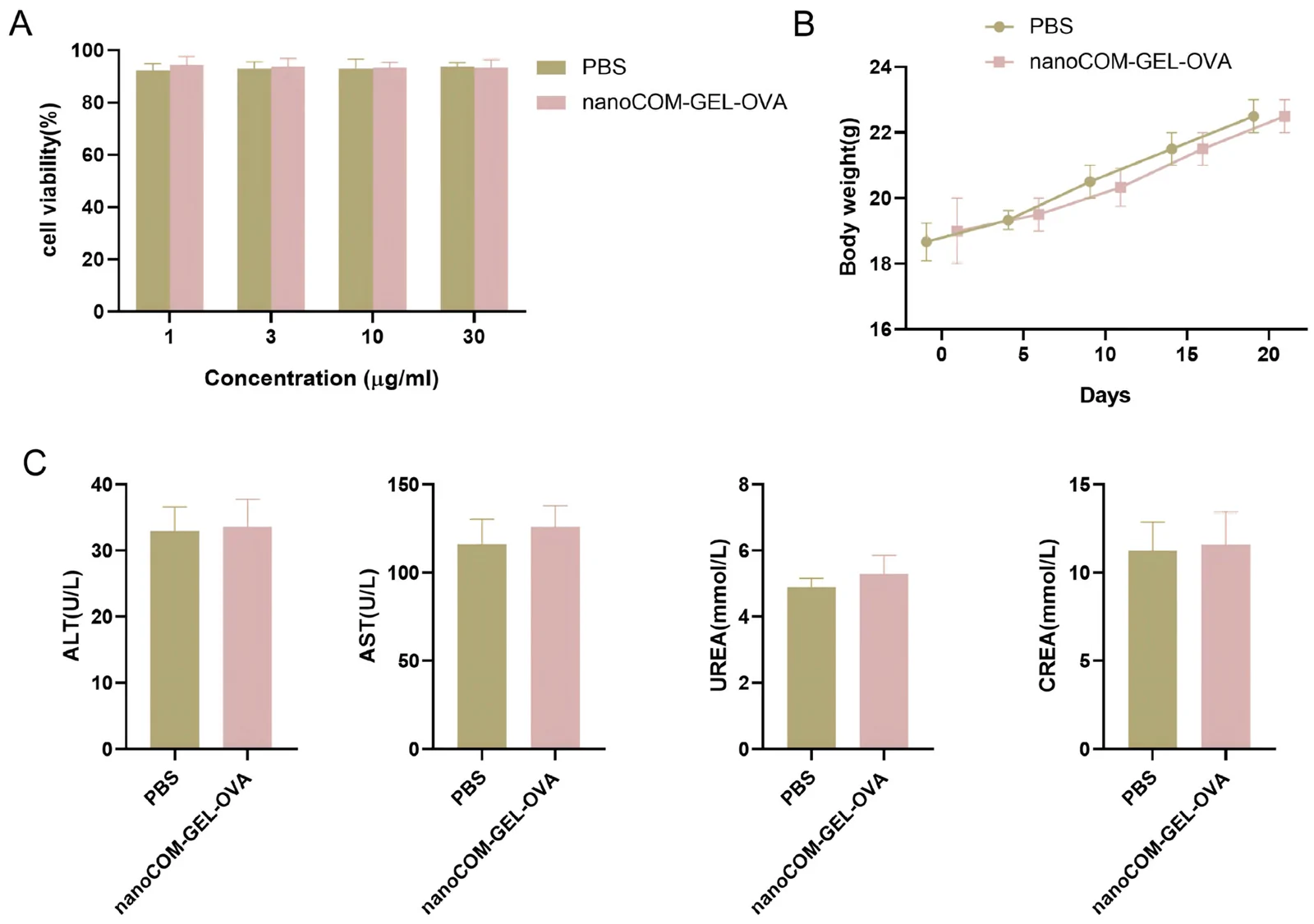
Biological safety of nanoCOM-GEL vaccine. (**A**) Viability of 293T cells following 24 h of exposure to escalating concentrations of nanoCOM-GEL-OVA. (**B**) Body weight changes observed in healthy C57BL/6 mice receiving intranasal doses of 50 μL nanoCOM-GEL-OVA (1 mg/mL) once every three days over a total of five consecutive administrations (*n* = 4 per group). (**C**) Levels of serum alanine aminotransferase (ALT), aspartate aminotransferase (AST), urea, and creatinine (CREA) measured in normal C57BL/6 mice after the same intranasal regimen. All quantitative data are presented as mean ± S.E.M. (*n* = 4). One-way ANOVA followed by Tukey’s multiple comparisons test was applied after confirming the assumptions of normality (Shapiro–Wilk test) and homogeneity of variance (Brown–Forsythe test).

The biosafety of nanoCOM-GEL is an essential step prior to any clinical application. To evaluate its cytotoxic potential, 293T cells were exposed to escalating concentrations of nanoCOM-GEL-OVA for 24 h. Even at the highest dose tested, nanoCOM-GEL did not produce any detectable cytotoxic effects (Figure 7A). Histological evaluation of the olfactory epithelium and brain (H&E staining) following repeated intranasal administration showed no signs of epithelial damage or inflammation (Figure S8A). Meanwhile, the mice intranasally administrated with nanoCOM-GEL-OVA maintained stable body weights throughout the observation period (Figure 7B). Moreover, key serum biochemical parameters (ALT, AST, CREA, and UREA) and inflammation parameters (IL-6 and IL-1β) remained comparable across all experimental groups, which confirmed that nanoCOM-GEL did not destroy liver or kidney function (Figure 7C and Figure S8B). Together, these results demonstrated that nanoCOM-GEL was biocompatible and could not induce overt toxicity upon intranasal delivery.

## 4. Discussion

In this study, we successfully constructed and validated nanoCOM-GEL, a next-generation therapeutic vaccine platform with the abilities of efficient antigen presentation, sustained release, efficient chemotaxis of immune cells, and tumor targeting. Through intranasal delivery, it overcame the physical barrier of the BBB, accumulated within the tumor, attracted immune cells to the tumor and reversed the immunosuppressive microenvironment. The nanoCOM-GEL vaccine induced robust tumoral and systemic antigen-specific immune responses and exhibited potent therapeutic efficacy against GBM.

The nanoCOM-GEL vaccine combines the advantages of GelMA and nanoparticles. In the human nasal cavity, olfactory mucosa accounts for less than 10% of the surface area and is subject to mucociliary clearance and the mucus barrier [35]. GelMA has RGD sequences, which enable it to adhere to mucosal epithelial cells and prevent mucociliary clearance. Using microfluidic technology, we controlled the particle size of nanoCOM-GEL to a stable range of 150–200 nm. This scale can precisely match the physiological window for nose-to-brain transport. Previous studies have shown that 100–200 nm particles can be efficiently transported to the brain via the intercellular spaces of the olfactory epithelium and perineural spaces [36,37,38,39]. Furthermore, the porous structure and minimum equilibrium volume expansion characteristics enable high-density encapsulation of peptide antigens, R848, CXCL16/MIP-1α and other components. In vitro release experiments demonstrated that the high encapsulation efficiency and the porous structure effectively prevented antigens from proteolytic degradation and prolonged the antigen half-life. These characteristics of nanoCOM-GEL are superior to those of conventional liposomal carriers and chitosan nanoparticles, which often suffer from rapid clearance and insufficient immunogenicity due to burst release effects [40]. Though a few recent publications have explored intranasal vaccination for GBM [11,41], none have combined the multi-component approach in a single hydrogel platform as we have done. Critically, in vitro degradation and release kinetics exhibited sustained and controllable degradation in simulated physiological conditions, laying the foundation for prolonged release.

Furthermore, the chemokines carried by nanoCOM-GEL could effectively recruit T cells and DCs to the tumor site. Migration assays demonstrated that nanoCOM-GEL not only served as a delivery vehicle for antigens but also functioned as a powerful immunocyte chemotaxis platform. By establishing a microenvironment favorable for attraction of DCs and T cells, it induced immune responses within the tumor. Intratumoral immunity can alter the local cytokine profile, reduce the proportion of suppressive cells, and reshape the immunosuppressive microenvironment [42]. In this study, we also confirmed that nanoCOM-GEL could induce M1 polarization of TAMs, which can also convert ‘cold tumors’ into ‘hot tumors’ and create favorable conditions for induction of anti-tumor immunity by vaccine [43]. Preclinical evidence across multiple tumor models has demonstrated that local delivery of immunostimulatory agents can synergize with systemic checkpoint blockade such as PD-1/PD-L1 and CTLA-4 by expanding the tumor-reactive T-cell pool while simultaneously removing the brakes on their cytotoxic activity [44,45]. Given that the nanoCOM-GEL system provides sustained local antigen release, it could serve as an ideal primer for the immune system, potentially converting “cold” tumors into “hot” tumors that are more susceptible to PD-1/PD-L1 or CTLA-4 blockade. Such a combinatorial strategy may not only increase the overall response rate but also reduce the required doses of ICIs, thereby mitigating their associated immune-related adverse events (irAEs). In this context, the combination of our vaccine platform with immune checkpoint inhibitors (ICIs) represents a rational and promising next step.

Several limitations of the present study should be acknowledged. First, the vaccine antigen used in this study was the model antigen ovalbumin (OVA), which, while enabling precise tracking of antigen-specific T-cell responses, possesses exceptionally high immunogenicity that may not accurately reflect the immune landscape of human glioblastoma. The therapeutic efficacy observed with OVA may therefore overestimate the effectiveness expected with endogenous glioma-associated antigens. Future studies incorporating clinically relevant antigens—such as EGFRvIII, IL13Rα2, survivin, WT1, or B7-H3-derived epitopes, as well as patient-specific neoantigens—will be essential to validate the translational potential of this platform. Second, while our intranasal delivery data demonstrate enhanced brain accumulation, we acknowledge that the current evidence does not definitively prove classical transcytosis across the BBB endothelium. Definitive mechanistic studies, such as LDLR blocking or integrin-mediated targeting assays, are needed. Third, we did not perform a more detailed characterization of regulatory T cells (FoxP3^+^), exhaustion markers (PD-1, TIM-3, LAG-3), NK cells, DC subsets, or memory T-cell responses. Such analyses would undoubtedly provide a more comprehensive picture of the vaccine-induced immune landscape. Furthermore, the long-term memory immunological effect of inhibiting tumor recurrence needs to be observed in future studies. The long-term biosafety of the vaccine also should be monitored. Additionally, the scalability and reproducibility of the nanoCOM-GEL fabrication process, the single-payload deletion experiment, as well as the long-term stability of the lyophilized formulation, need to be systematically validated under good manufacturing practice (GMP) conditions.

## 5. Conclusions

In conclusion, we used GelMA as the hydrogel matrix material to synthesize a composite hydrogel nanovaccine (nanoCOM-GEL-OVA) for GBM therapy by incorporating a BBB-penetrating peptide, immune cell stimulants, chemokines, and antigenic peptides. The nanoCOM-GEL vaccine exhibited high encapsulation efficiency and controlled release properties. Following intranasal immunization, it could enter the brain via the olfactory bulb. Moreover, through the RGD motif inherent to GelMA, the nanoCOM-GEL accumulated within the GBM and induced intratumoral immunity. Intranasal immunization of nanoCOM-GEL-OVA effectively elicited potent anti-GL261-OVA-luc immune responses, activated intracranial tumor antigen-specific T cells, reversed the immunosuppressive microenvironment of GBM and suppressed the growth of GL261-OVA-luc cells. Meanwhile, the nanoCOM-GEL-OVA showed no evident short-term toxicity under the tested conditions. This novel nanocomposite hydrogel vaccine provides a new platform for GBM immunotherapy via the nose-to-brain delivery route. This study lays the preclinical foundation for the translational application of this novel nanocomposite hydrogel vaccine.

## Data Availability

The original contributions presented in this study are included in the article/Supplementary Material. Further inquiries can be directed to the corresponding authors.

## Supplementary Materials

The following supporting information can be downloaded at: https://www.mdpi.com/article/10.3390/vaccines14090797/s1, Table S1: The various nanohydrogels used in this study and their compositions; Table S2: Antibody information; Table S3: Primers for genes; Figure S1: Encapsulation efficiency (%) of OVA peptide, peptide 22, R848, CXCL16 and MIP-1α in nanoCOM-GEL. Figure S2: The ability of the nanoCOM-GEL vaccine on macrophage polarization invitro; Figure S3: The binding of nanogel synthesized with GelMA to integrin αvβ3 on GBM was inhibited by anti-αvβ3 antibody; Figure S4: Proliferation ability of OVA specific T cells in tumor (A), spleen (B) and lymph node (C) from mice treated with different nano hydrogal vaccines; Figure S5: The effects of nanoCOM-GEL-OVA vaccines on DCs infiltration in tumor; Figure S6: The ability of the nanoCOM-GEL vaccine on macrophage polarization in vivo; Figure S7: The effects of nanoCOM-GEL-OVA vaccines on serum cytokines; Figure S8: Biological safety of nanoCOM-GEL vaccine.

## Abbreviations

The following abbreviations are used in this manuscript:

GBM	glioblastoma
BBB	blood–brain barrier
TAM	tumor-associated macrophages
GelMA	gelatin methacryloyl
VLP	virus-like particles
GFAP	glial fibrillary acidic protein
CR	complete remission
OVA	ovalbumin
TME	Tumor immunosuppressive microenvironment
MDSC	myeloid-derived suppressor cells
SEM	scanning electron microscopy
TEM	transmission electron microscope
DAB	3,3′-diaminobenzidine
ALT	alanine aminotransferase
AST	aspartate aminotransferase
CREA	creatinine
IVIS	in vivo imaging system
APC	antigen-presenting cells
BBTB	blood–brain tumor barrier
