# Supplementary material for: Nanohydrogel Composite Vaccine Capable of Bypassing the Blood–Brain Barrier and Targeting Tumors for Glioblastoma Immunotherapy via Intranasal Immunization
Source: Vaccines (Basel). 2026 Sep 10;14(9):797. doi: 10.3390/vaccines14090797 (PMC13611568; doi:10.3390/vaccines14090797)
Supplement: Supplementary file 1 [file vaccines-14-00797-s001.zip › vaccines-4438868-supplementary.pdf]

Supplementary Information

Materials and Methods

Table S1　The various nanohydrogels used in this study and their compositions.

Gel Formulation	OVA Peptide	Peptide 22	R848	MIP-1α	CXCL16
nanogel	✗	✗	✗	✗	✗
nanogel-OVA	✓	✗	✗	✗	✗
nanogel-pep22	✗	✓	✗	✗	✗
nanogel-pep22-OVA	✓	✓	✗	✗	✗
nanoCOM-GEL	✗	✓	✓	✓	✓
nanoCOM-GEL-OVA	✓	✓	✓	✓	✓

Table S2　Antibodies information

Antibody	Conjugate	Brand	Catalog Number
αvβ3 integrin	Unconjugated	Abcam	ab190147
CD11c	PerCP-Cy5.5	Thermo Fisher	45-0114-82
CD80	FITC	Thermo Fisher	A14722
CD40	APC	Thermo Fisher	17-0402-82
CD86	FITC	Thermo Fisher	MA1-10300
MHC II (I-A/I-E)	APC	Thermo Fisher	17-5320-82
CD3	FITC	Thermo Fisher	MHCD0301
CD8	PE	Thermo Fisher	MA1-19626
IFN-γ	APC	Thermo Fisher	47-7319-42

Antibody	Conjugate	Brand	Catalog Number
F4/80	Alexa Fluor™ 647	Thermo Fisher	MF48021
NK1.1	PE-Cy7	Thermo Fisher	25-5941-81

Table S3 Primers for genes

Genes	Forward Primer	Reverse Primer
IL-12	GGAAGCACGGCAGCAGCAGAATA	AACTTGAGGGAGAAGTAGGAATGG
iNOS	CCTTGTTCAGCTACGCCTTC	GGTATGCCCGAGTTCTTTCA
TNF- α	CATCTTCTCAAAATTCGAGTGACAA	TGGGAGTAGACAAGGTACAACCC
TGF- β	ACAGGGCTTTCGATTCAGCG	CACTTGCAGGAGCGCACAAT
IL-10	TGCCTTCAGCCAGGTGAAGACT	AAATTCATTCATGGCCTTGTA

Real-time quantitative PCR analysis:

Total RNA was extracted from cultured cells or homogenized tissue samples using TRIzol® Reagent (Invitrogen, Carlsbad, CA, USA) according to the manufacturer's instructions. RNA concentration and purity were assessed by measuring the absorbance at 260 nm and 280 nm using a NanoDrop™ spectrophotometer (Thermo Fisher Scientific, USA), and only samples with an A_{260}/A_{280} ratio between 1.8 and 2.0 were used for subsequent analysis. First-strand cDNA was synthesized from 1 μg of total RNA using the PrimeScript™ RT Master Mix (Takara Bio, Japan) in a 20 μL reaction volume, with the following thermal profile: 37°C for 15 min for reverse transcription, followed by 85°C for 5 s to inactivate the enzyme. The resulting cDNA was then diluted 1:5 with nuclease-free water and stored at −20°C until use. Quantitative real-time PCR was performed using TB Green® Premix Ex Taq™ II (Takara Bio, Japan) on a ABI 7500 Real-Time PCR System (ABI, Switzerland). Each 20 μL reaction mixture contained 10 μL of TB Green Premix, 2 μL of diluted cDNA, 0.8 μL each of forward and reverse primers (final concentration 0.4 μM), and 6.4 μL of nuclease-free water. The PCR cycling conditions were as follows: initial denaturation at 95°C for 30 s, followed by 40 cycles of 95°C for 5 s and 60°C for 30 s. A melting curve analysis was performed at the end of each run to verify the specificity of the amplified products, with a temperature gradient from 65°C to 95°C at 0.1°C/s. The relative expression levels of target genes were calculated using the $2^{-\Delta\Delta Ct}$ method, with GAPDH (for mouse samples) or β-actin (for human samples) as the internal reference gene. All reactions were performed in

triplicate, and the results were expressed as fold-change relative to the control group. The primer sequences used for each target gene are listed in Supplementary Table S2.

Encapsulation efficiency determination:

To determine the encapsulation efficiency (EE) of R848, MIP-1α, and CXCL16 in the nanoCOM-GEL formulation, a solvent extraction method combined with ultrafiltration centrifugation was employed. Briefly, 500 μL of the freshly prepared nanoCOM-GEL suspension was transferred into an ultrafiltration centrifuge tube (molecular weight cut-off: 10 kDa, Millipore, USA) and centrifuged at 4,000 × g for 30 min at 4°C to separate the encapsulated payload from the unencapsulated free fraction. The filtrate containing the free (unencapsulated) R848, MIP-1α, and CXCL16 was collected for quantification. To determine the total amount of each compound in the hydrogel, an equivalent volume of the nanoCOM-GEL suspension was thoroughly disrupted by adding 1 mL of acetonitrile (for R848) or lysis buffer (for cytokines), followed by vortexing and sonication for 10 min to completely release the encapsulated cargo. The resulting solution was then centrifuged at 12,000 × g for 10 min, and the supernatant was collected. For R848 quantification, the supernatant was analysed using high-performance liquid chromatography (HPLC) with a C18 column and UV detection at 325 nm, with a mobile phase consisting of acetonitrile and 0.1% trifluoroacetic acid in water (40:60, v/v) at a flow rate of 1.0 mL/min. For MIP-1α and CXCL16 quantification, the supernatants and ultrafiltrates were analysed using commercially available enzyme-linked immunosorbent assay (ELISA) kits (R&D Systems, USA) according to the manufacturer's protocols. All samples were measured in triplicate. The encapsulation efficiency (EE%) for each component was calculated using the formula: EE% = [(Total amount – Free amount) / Total amount] × 100%. The method was validated for recovery, linearity, and precision, and the results were expressed as mean ± SD from three independent experiments.

Preparation of chitosan-based nanohydrogel:

The chitosan-based nanohydrogel was prepared via an ionic crosslinking and microemulsion method. Briefly, chitosan (medium molecular weight, 50–190 kDa, deacetylation degree ≥ 75%, Sigma-Aldrich, USA) was dissolved in 1% (v/v) acetic acid solution at a concentration of 10 mg/mL under continuous stirring at room temperature overnight to obtain a homogeneous chitosan stock solution. The pH of the solution was then adjusted to 5.5 using 1 M NaOH. Separately, the payload components—including Peptide 22 (10 μg/mL), R848 (5 μg/mL), recombinant mouse MIP-1α (100 ng/mL), and CXCL16 (100 ng/mL)—were dissolved in sterile phosphate-buffered saline (PBS, pH 7.4) and mixed with the chitosan solution under gentle magnetic stirring at 500 rpm for 30 min at 4°C to form the aqueous phase. To prepare the nanohydrogel, tripolyphosphate (TPP, 0.5 mg/mL in distilled water, pH 8.0) was used as the ionic crosslinker and was added dropwise to the chitosan/payload mixture under constant stirring at 800 rpm, at a chitosan:TPP mass ratio of 5:1, and the reaction was allowed to proceed for 1 hour at room temperature. The resulting nano-sized hydrogel particles were then purified by centrifugation at 10,000 × g for 15 min at 4°C, washed three times with distilled water to remove unencapsulated components and residual TPP, and resuspended in PBS (pH 7.4) at a final chitosan concentration of 5 mg/mL.

Cy 2 and Cy5.5 labeling:

NanoCOM-GEL was labeled with Cy5.5-NHS ester (Lumiprobe, USA). Briefly, Cy5.5-NHS ester (1 mg/mL in DMSO) was added to the nanogel dispersion at a final concentration of 10 μg/mg nanogel. The mixture was incubated at 4°C for 4 hours in the dark. Unbound Cy5.5 was removed by dialysis against PBS (MWCO 10 kDa) for 24 hours with 3 buffer changes. The labeled nanogel was stored at 4°C in the dark until use.

Ex vivo detection of Cy2-labelled nanoCOM-GEL biodistribution:

To evaluate the biodistribution of Cy2-labelled nanoCOM-GEL, C57BL/6 mice (n = 3 per time point) were administered with Cy2-labelled nanoCOM-GEL via intranasal administration. At designated time points (1 h, 2 h and 4h post-administration), the mice were humanely euthanised and transcardially perfused with 20 mL of ice-cold PBS followed by 20 mL of 4% paraformaldehyde (PFA) in PBS (pH 7.4) to remove blood and fix the tissues. Major organs including the brain, heart, liver, spleen, lung and kidney were carefully harvested, the Cy2 signal was observed by IVIS. Furthermore, the brain was post-fixed in 4% PFA at 4°C for 24 hours, and then cryoprotected in 30% sucrose (w/v) in PBS at 4°C for 48 hours until the tissues sank to the bottom. The tissues were then embedded in OCT compound (Sakura, USA), frozen at −80°C, and sectioned into 10 μm thick coronal slices using a cryostat (Leica CM3050S, Germany). The tissue sections were mounted onto poly-L-lysine-coated glass slides, air-dried, and washed with PBS to remove the OCT compound. Nuclei were counterstained with DAPI (4′,6-diamidino-2-phenylindole, 1 μg/mL, Sigma-Aldrich, USA) for 10 min at room temperature, and the sections were mounted with an anti-fade mounting medium. Fluorescence images were acquired using a whole-slide scanner (3DHISTECH Pannoramic SCAN II) .

Intravenous administration of nanoCOM-GEL in mice:

To evaluate the systemic biodistribution and potential toxicity of the nanoCOM-GEL formulation, C57BL/6 mice (6–8 weeks old, 18–22 g) were administered with nanoCOM-GEL via intravenous injection. Briefly, the nanoCOM-GEL suspension was freshly prepared in sterile phosphate-buffered saline (PBS, pH 7.4) at a concentration of 5 mg/mL and gently vortexed to ensure uniform dispersion. Prior to injection, mice were placed in a warming box (37°C) for 5–10 minutes to dilate the tail veins. Each mouse received a single bolus injection of nanoCOM-GEL (100 μL, containing 500 μg of hydrogel) into the lateral tail vein using a 30-gauge insulin syringe. The injection site was gently pressed with sterile gauze for 30 seconds immediately after needle withdrawal to prevent bleeding.

Cytokine quantification by ELISA:

To quantify the levels of IFN-γ, IL-12p70, TNF-α, IL-1β and IL-6 in serum, commercially available enzyme-linked immunosorbent assay (ELISA) kits were used according to the manufacturers' protocols (ExCell bio, Shanghai, China). Briefly, 96-well microtiter plates pre-coated with capture antibodies specific for each cytokine were incubated with 100 μL of samples or serial dilutions of recombinant cytokine standards at 4°C overnight. After washing three times with PBS-T (0.05% Tween-20 in PBS), 100 μL of biotinylated detection antibody was added to each well and incubated for 2 hours at room temperature. The plates were then washed, followed by the addition of streptavidin-horseradish peroxidase (HRP) conjugate for 20–30 min in the dark. After a final wash, 100 μL of tetramethylbenzidine (TMB) substrate solution was added, and the plates were incubated at room temperature for 15–30 min until colour development was observed. The reaction was stopped by adding 50 μL of 2 M H_2SO_4, and the absorbance was measured at 450 nm using a microplate reader (BioTek, USA), with wavelength correction at 570 nm. Cytokine concentrations were calculated by interpolation from the standard curve generated by four-parameter logistic (4-PL) regression analysis using GraphPad Prism software (version 8.0). All samples were measured in duplicate.

Figures

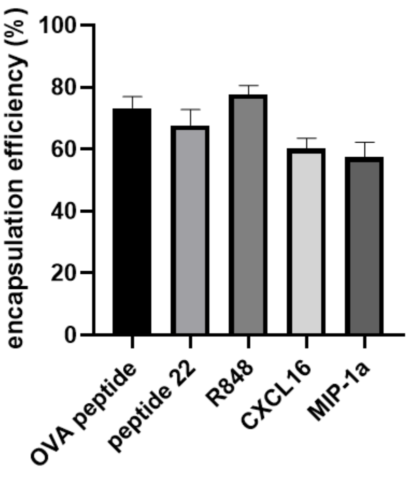

Figure S1 Encapsulation efficiency (%) of OVA peptide, peptide 22, R848, CXCL16 and MIP-1α in nanoCOM-GEL.

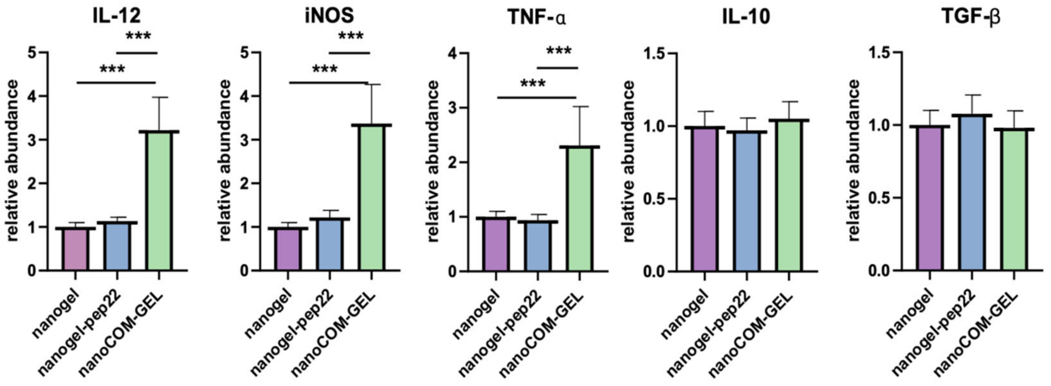

Figure S2 The ability of nanoCOM-GEL vaccine on macrophage polarization *invitro*. After 48 h co-treatment of primary macrophages with IL-4/IL-13 and nanoCOM-GEL, the expression of M1/M2 related genes by RT-PCR. Data represent mean ± S.E.M. n=3. One-way ANOVA followed by Tukey's multiple comparisons test was applied, after confirming the assumptions of normality (Shapiro–Wilk test) and homogeneity of variance (Brown–Forsythe test). ***$p < 0.001$.

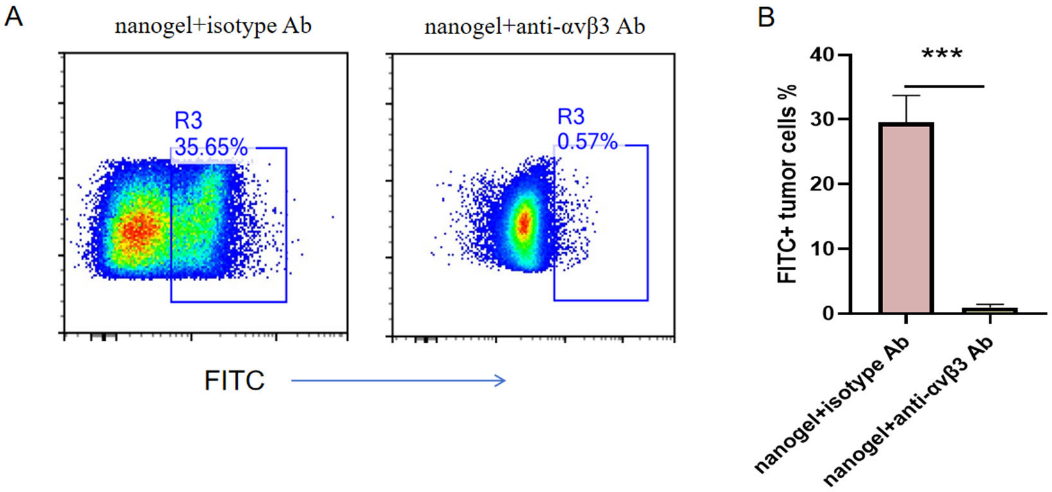

Figure S3 The binding of nanogel synthesized with GelMA to integrin αvβ3 on GBM was inhibited by anti-αvβ3 antibody. Representative flow cytometry images showing the nanogel synthesized with GelMA in binding glioblastoma cell line in the presence of anti-αvβ3 antibody**(A)** and quantitative analysis**(B)**. Data represent mean ± S.E.M. n=3. One-way ANOVA followed by Tukey's multiple comparisons test was applied, after confirming the assumptions of normality (Shapiro–Wilk test) and homogeneity of variance (Brown–Forsythe test). ***, $p<0.001$;

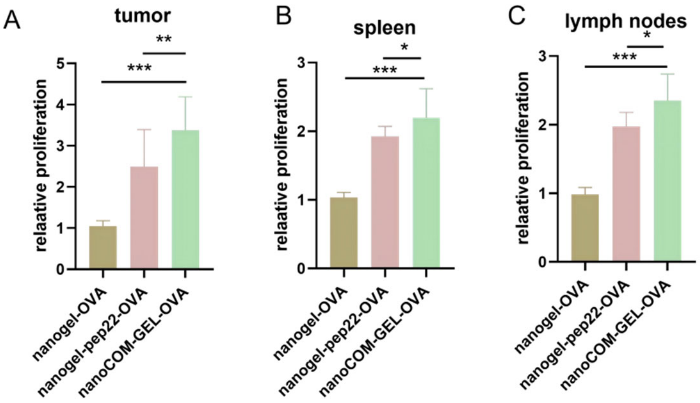

Figure S4. Proliferation ability of OVA specific T cells in tumor(A) , spleen(B) and lymph node(C) from mice treated with different nano hydrogal vaccines. Data represent mean ± S.E.M. n=3. One-way ANOVA followed by Tukey's multiple comparisons test was applied, after confirming the assumptions of normality (Shapiro–Wilk test) and homogeneity of variance (Brown–Forsythe test)., *$p < 0.05$, **$p < 0.01$, ***$p < 0.001$.

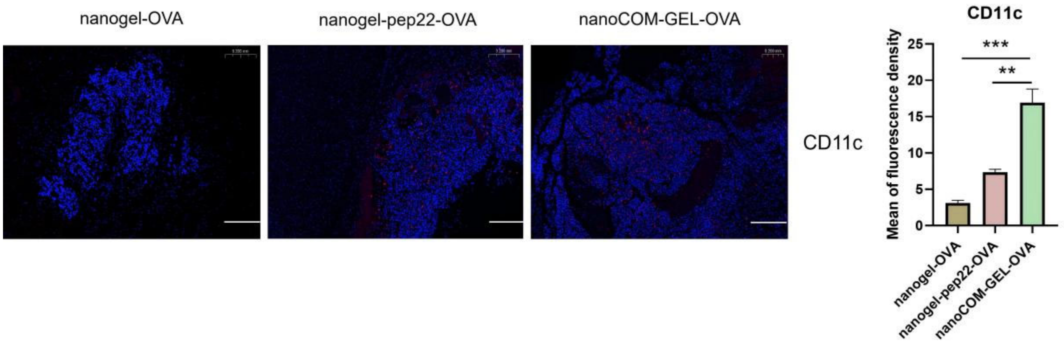

Figure S5. The effects of nanoCOM-GEL-OVA vaccines on DCs infiltration in tumor. The images of immunofluorescence staining of brain slices after mice intranasally administrated with various nanohydrogel as indicated in the figure and quantitative analysis. Red: CD11c; blue: nucleus stained by DAPI. Scale bars: 50 μm. Data represent mean ± S.E.M. n=3. One-way ANOVA followed by Tukey's multiple comparisons test was applied, after confirming the assumptions of normality (Shapiro–Wilk test) and homogeneity of variance (Brown–Forsythe test). **p < 0.01, ***p < 0.001.

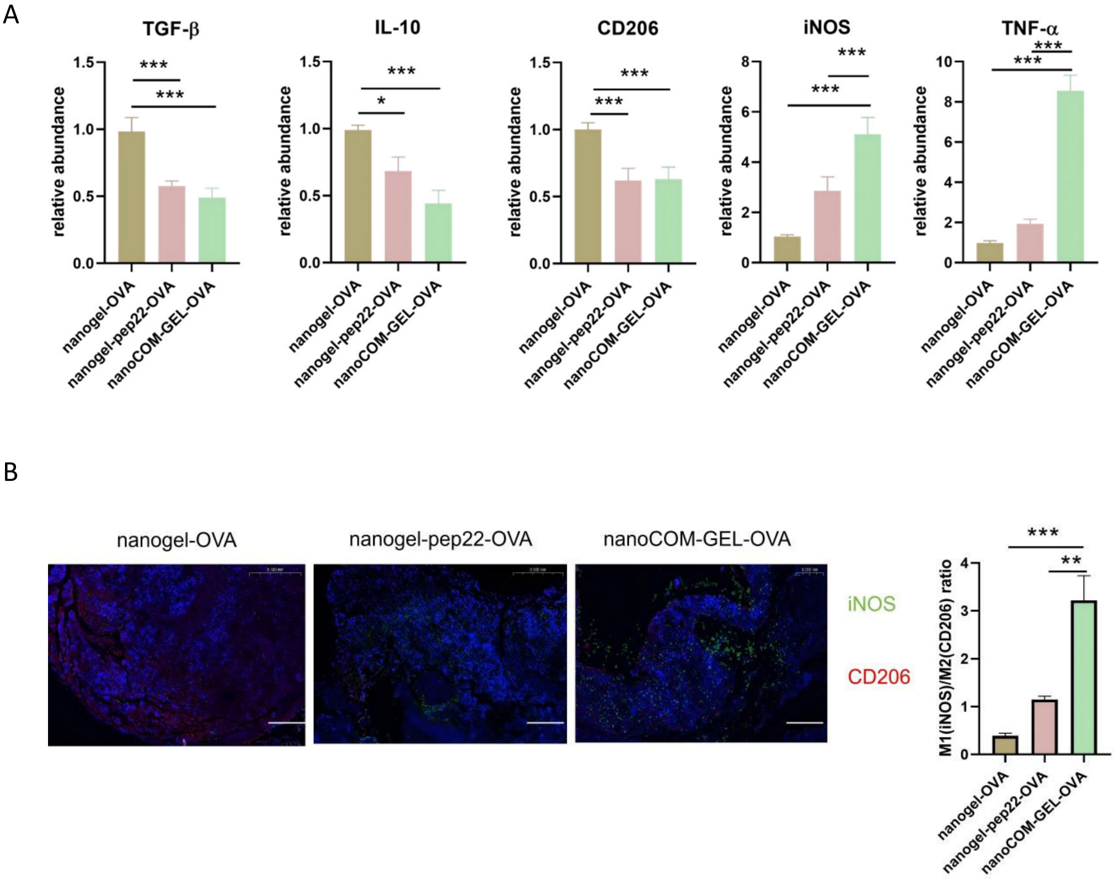

Figure S6. The ability of nanoCOM-GEL vaccine on macrophage polarization *invivo*. (A) Three days after intranasal immunization of nanoCOM-GEL in GBM bearing mice, the TAMs were isolated and detected the expression of M1/M2 related genes by RT-PCR. **(B)** The images of immunofluorescence staining of brain slices after mice intranasally administrated with various nanohydrogel as indicated in the figure and quantitative analysis. Red: CD206; green: iNOS; blue: nucleus stained by DAPI. Scale bars: 50 μm. Data represent mean ± S.E.M. n=3. One-way ANOVA followed by Tukey's multiple comparisons test was applied, after confirming the assumptions of normality (Shapiro–Wilk test) and homogeneity of variance (Brown–Forsythe test). *p < 0.05, ***p < 0.001

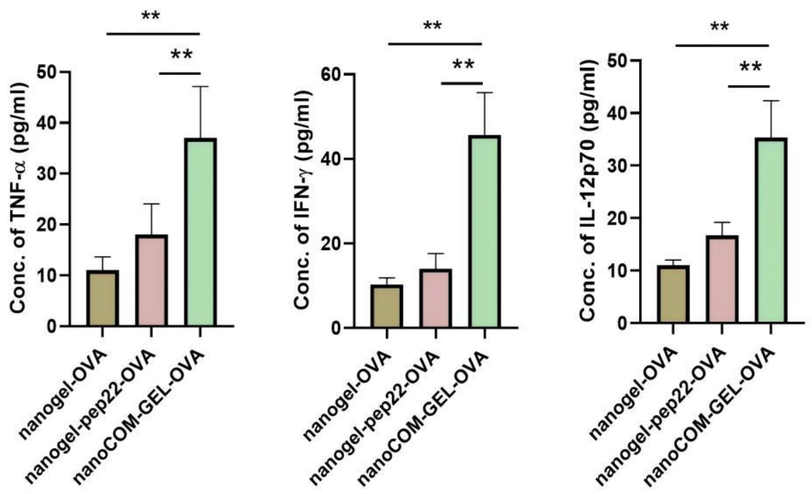

Figure S7. The effects of nanoCOM-GEL-OVA vaccines on serum cytokines . Three days after intranasal immunization of nanoCOM-GEL in GBM bearing mice, the serum were collected and the cytokines concentration in serum were detected by ELISA. Data represent mean ± S.E.M. n=3. One-way ANOVA followed by Tukey's multiple comparisons test was applied, after confirming the assumptions of normality (Shapiro–Wilk test) and homogeneity of variance (Brown–Forsythe test). **p < 0.01.

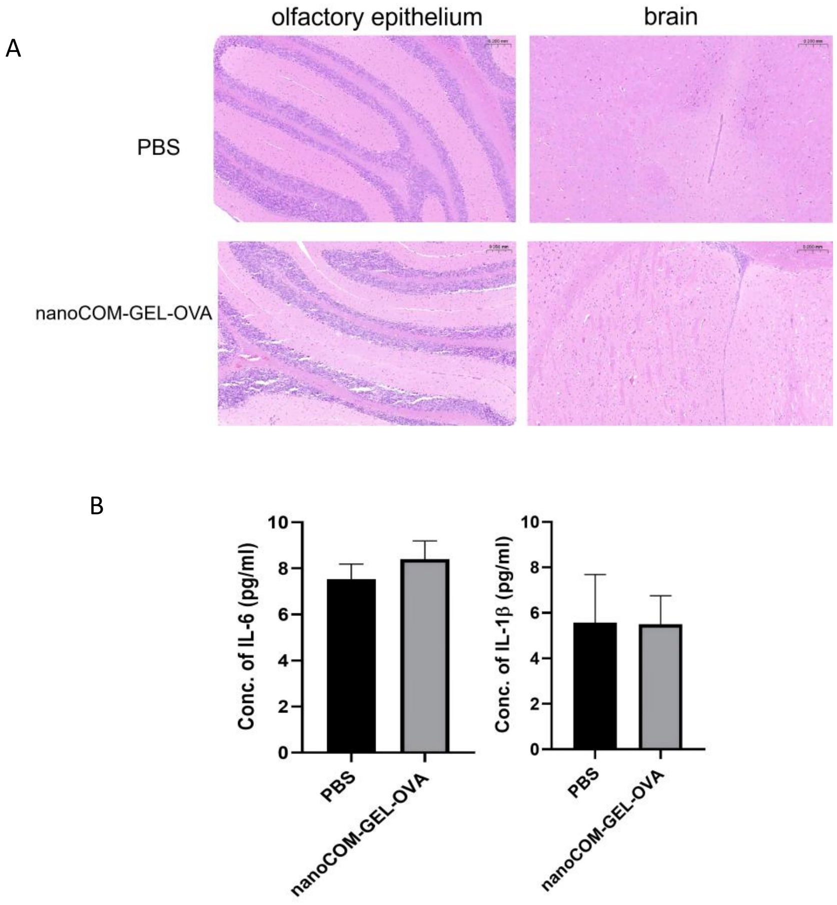

Figure S8 Biological safety of nanoCOM-GEL vaccine. (A) H&E staining of brain and olfactory epithelium of normal C57BL/6 mice after the same intranasal administration of PBS and nanoCOM-GEL-OVA. (B) The serum concentration of IL-6 & IL-1β in normal C57BL/6 mice after the same intranasal administration of PBS and nanoCOM-GEL-OVA. Data represent mean ± S.E.M. n=3. One-way ANOVA followed by Tukey's multiple comparisons test was applied, after confirming the assumptions of normality (Shapiro–Wilk test) and homogeneity of variance (Brown–Forsythe test).
